# Characterisation of *Staphylococcus aureus* Strains and Their Prophages That Carry Horse-Specific Leukocidin Genes *lukP/Q*

**DOI:** 10.3390/toxins17010020

**Published:** 2025-01-03

**Authors:** Stefan Monecke, Sindy Burgold-Voigt, Andrea T. Feßler, Martina Krapf, Igor Loncaric, Elisabeth M. Liebler-Tenorio, Sascha D. Braun, Celia Diezel, Elke Müller, Martin Reinicke, Annett Reissig, Adriana Cabal Rosel, Werner Ruppitsch, Helmut Hotzel, Dennis Hanke, Christiane Cuny, Wolfgang Witte, Stefan Schwarz, Ralf Ehricht

**Affiliations:** 1 Leibniz Institute of Photonic Technology (Leibniz-IPHT), Leibniz Center for Photonics in Infection Research (LPI), Germany and InfectoGnostics Research Campus, 07745 Jena, Germany; 2 Institute of Microbiology and Epizootics and Veterinary Centre for Resistance Research (TZR), School of Veterinary Medicine, Freie Universität Berlin, 14163 Berlin, Germany; 3 LABOKLIN GmbH&Co. KG, 97688 Bad Kissingen, Germany; 4 Institute of Microbiology, University of Veterinary Medicine Vienna, 1210 Vienna, Austria; 5 Institute of Molecular Pathogenesis, Friedrich-Loeffler-Institute (Federal Research Institute for Animal Health), 07743 Jena, Germany; 6 Austrian Agency for Health and Food Safety (AGES), Institute for Medical Microbiology and Hygiene, 1090 Vienna, Austria; 7 FoodHub—Centre of Excellence for Digitalisation of Microbial Food Safety Risk Assessment and Quality Parameters for AccurFood Authenticity Certification, University of Donja Gorica, 81000 Podgorica, Montenegro; 8 Institute of Bacterial Infections and Zoonoses, Friedrich-Loeffler-Institute (Federal Research Institute for Animal Health), 07743 Jena, Germany; 9 Robert Koch Institute, Wernigerode Branch, 38855 Wernigerode, Germany; 10 Institute of Physical Chemistry, Friedrich-Schiller University, 07743 Jena, Germany

**Keywords:** *Staphylococcus aureus*, leukocidins, prophages, bacteriophages, *lukP/Q*, horse (*Equus caballus*), whole-genome sequencing, DNA-microarrays, electron microscopy

## Abstract

Leukocidins of *Staphylococcus* (*S*.) *aureus* are bicomponent toxins that form polymeric pores in host leukocyte membranes, leading to cell death and/or triggering apoptosis. Some of these toxin genes are located on prophages and are associated with specific hosts. The genes *lukP/Q* have been described from equine *S. aureus* isolates. We examined the genomes, including the *lukP/Q* prophages, of *S. aureus* strains belonging to clonal complexes CC1, CC350, CC816, and CC8115. In addition to sequencing, phages were characterised by mitomycin C induction and transmission electron microscopy (TEM). All *lukP/Q* prophages integrated into the *lip2*=*geh* gene, and all included also the gene *scn-eq* encoding an equine staphylococcal complement inhibitor. The *lukP/Q* prophages clustered, based on gene content and allelic variants, into three groups. One was found in CC1 and CC97 sequences; one was present mainly in CC350 but also in other lineages (CC1, CC97, CC133, CC398); and a third one was exclusively observed in CC816 and CC8115. Prophages of the latter group additionally included a rare enterotoxin A allele (*sea*_320E_). Moreover, a prophage from a CC522 goat isolate was found to harbour *lukP*. Its *lukF* component could be regarded as chimaera comprising parts of *lukQ* and of *lukF-P83*. A putative kinase gene of 1095 basepairs was found to be associated with equine strains of *S. aureus*. It was also localised on prophages. However, these prophages were different from the ones that carried *lukP/Q*, and three different integration sites of kinase-carrying phages were identified. These observations confirmed the presence of prophage-located important virulence-associated genes in equine *S. aureus* and that certain prophages might determine the host specificity of the staphylococcal strains they reside in.

## 1. Introduction

*Staphylococcus aureus* is a globally spread bacterium that colonises the nasal cavities of about 20–30% of any human population and a considerable percentage of wild and domestic mammal and bird populations [1]. Beyond colonisation, *S. aureus* is also able to cause a wide spectrum of clinical infections that range from superficial skin and soft tissue infections (SSTI) to life-threatening conditions, such as sepsis, endocarditis, necrotising fasciitis, and pneumonia. *S. aureus* has become resistant, by acquisition and exchange of mobile genetic elements, to numerous anti-infective compounds. The most relevant examples are so-called SCC*mec* elements that harbour *mec* genes, which gave rise to a number of genetically diverse methicillin/beta-lactam-resistant *S. aureus* strains, or MRSA [2,3,4,5,6,7].

Among the virulence factors of *S. aureus*, there is a staggering array of adhesion- and biofilm-related factors, superantigenic toxins, haemolysins, and leukocidins. The latter are bicomponent proteins that consist of two different molecules, encoded by co-localised and co-expressed genes, that form polymeric pores in host leukocyte membranes, resulting in cell death or apoptosis [8,9,10]. Some leukocidins (Supplemental File S1) can be found in virtually all *S. aureus*. These include *lukA/B* ([11,12], also known as *lukG/H* [13], or *lukX/Y* [7]; locus tags SACOL2004/SACOL2006 in CP000046.1) and *lukS/F* (SACOL2421/SACOL2422 in CP000046.1) from the haemolysin gamma locus. Another leukocidin is encoded by *lukE/D* (SACOL1881/SACOL1880 in CP000046.1), located on a genomic island (GI). Finally, there are phage-borne leukocidin genes. Due to the mobility of phages, they can be found across unrelated lineages. Panton-Valentine leukocidin (PVL), encoded by *lukS/F-PV*, belongs to that class. It is frequently detected in isolates from humans and is strongly associated with SSTIs, such as abscesses and carbuncles, but it can also cause necrotising pneumonia [14,15,16,17,18,19,20,21,22,23,24]. Similar genes, *lukS/F-BV*, have recently been described in isolates from moribund or dead beavers (*Castor fiber*) [25]. Other phage-borne leukocidin genes, *lukM/lukF-P83* [26,27,28], are regionally common in isolates from cattle, small ruminants, or, in rare cases, from pigs. These genes also have been associated with dermatitis in tree squirrels (*Sciurus vulgaris*) [29,30]. Finally, *lukP/Q* genes have recently been observed in a phage derived from horse isolates [31].

These genes are most closely related to the GI-encoded *lukE/D* genes, with gene products sharing 91% (LukP to LukE) and 80% (LukQ to LukD) amino acid sequence identities, respectively. LukP/Q preferentially kills equine neutrophils, with higher efficiency than LukE/D [31]. LukP/Q targets equine CXCR1 and CXCR2 receptors that mediate neutrophil accumulation at sites of inflammation and infection. It also shows some activity against bovine and human neutrophils, indicating a broad host range [31].

Interestingly, a second virulence factor, *scn-eq* (encoding, equine staphylococcal complement inhibitor, eqSCIN), is located on the same prophage. This is a potent inhibitor of complement activation and phagocytosis in equine serum, blocking the deposition of C3b on bacterial surfaces by inhibition of the C3 convertase. It can, to a lesser extent, also inhibit complement activation in human and porcine sera [32].

Horses have indeed frequently been observed to carry *S. aureus*, so that “horse-specific” virulence factors in *S. aureus* are of high interest. When reviewing available literature [33,34,35] and published genome sequences of horse isolates (*n* = 146, see https://www.bv-brc.org/view/Taxonomy/1279#view_tab=genomes&filter=and(eq(host_common_name,%22Horse%22),eq(genome_quality,%22Good%22),keyword(aureus)), accessed on 6 June 2024), one might discern different groups of lineages and strains that colonise or infect horses.

A first group of *S. aureus* in horses are obviously human-associated strains that have been transmitted to horses either directly from human contacts or via veterinary hospitals. These strains are often MRSA and frequently carry virulence markers known to be specific for humans, such as PVL. Many, but not all, of them belong to the CC8 as defined by multilocus sequence typing (MLST). In the USA, horses have been found with the notorious community-acquired, PVL-positive “USA300” strain (GenBank accession numbers JAAFNB01, JAAFMS01, JAAFMR01, JAAFMO01, JAAFMF01). In Germany, many horse isolates of the “Hannover epidemic strain”, CC8, sequence type (ST) 254, have been found. This was a common hospital-acquired MRSA strain in the 1990s [36]. In Australia, there was a curious outbreak of a CC8/ST612 strain that was imported from South Africa, supposedly by expatriate veterinarians or medical staff. Its spread was aided by veterinary use of antimicrobial compounds (trimethoprim/sulfamethoxazole and rifampicin), to which the strain acquired resistance in South Africa, where they are frequently used in human medicine [35]. Other human-associated lineages that sporadically were found in horses include CC6, CC22, CC45, and CC188 (see Supplemental File S2a), as well as CC15 [37].

A second group of horse strains are livestock-associated strains. *S. aureus* CC398 (including ST398, ST6239, see Supplemental File S2a and [37]), with or without SCC*mec* elements and *mecA*, is a most prominent example. These strains are, in the Western world, livestock-associated and frequently found in pigs, turkeys, and chickens, but also in personnel of farms and abattoirs [38,39,40,41,42,43,44]. CC398 might be transmitted to horses by farm or veterinary staff, but also due to close proximity to livestock, by exposure to faecal matter on pasture land, to dust and aerosols, or to flies. Other livestock-associated lineages observed in horses are CC97, which occurs in cattle and cervids, and CC133, which is frequently found in small ruminants, such as goats and sheep [45].

Third, there are lineages that can be found in different species, but in which horizontally transferred genes determine the host specificity of a given isolate. In such lineages, PVL- or *sak/chp/scn*-positive strains occur in humans while *lukP/Q*-positive ones are native to horses. CC1 is one example of such a lineage. It is widespread and common in humans, but isolates can also be found in horses, where they tend to carry *lukP/Q* genes. Similarly, *lukP/Q-*positive CC350 strains (which include ST1660 as well as the recently described ST6238 [37,46]) appear to be common in horses, while *lukP/Q*-negative isolates might be found in humans.

Finally, some sporadic lineages have been found in horses about which information is insufficient, so it is unknown whether they are native to horses, humans, or any other mammals. Examples are CC816 (see below) or CC4215 (SAMN13948107), as well as, possibly, ST2679/ST6242 [37], ST7636 [46], and CC8115 (see below).

In the present manuscript, we aim at characterising phages that carry horse-specific virulence factors and discuss and review their distribution and molecular features. We describe four *lukP/Q* carrying *S. aureus* lineages (CC1, CC350, CC816, and CC8115) in detail, the latter two of which have not yet been described. We obtained long-read (Nanopore) sequences for representative isolates of each of these four CCs and compared them, where possible, to additional short-read (Illumina) sequences of equine isolates of *S. aureus* either generated by the authors of this study or published previously.

## 2. Results

### 2.1. lukP/Q-Positive CC1-MSSA Isolates and Sequences

Isolate IMT39173 (Nanopore sequence, GenBank CP176568; Illumina sequence, VSZG01) belonged, as all CC1 isolates do, to *agr* group III and capsule type 8. Isolate IMT39173 had the enterotoxin H gene *seh*; it carried haemolysin/leukocidin genes *hlgA/lukS/lukF*, *lukE/D*, and *lukA/B*, and regarding CC-associated adhesion factors, it was positive for *cna* and *sasG* genes (that code for collagen adhaesin and *S. aureus* surface protein G, respectively). Thus, it was a typical representative of CC1.

Contrarily to sequences of human-derived CC1 strains (such as GenBank BX571857, BA000033), it also carried the equine leukocidin genes *lukP/Q* and the equine staphylococcal complement inhibitor gene *scn-eq* [32], as well as a prophage with a putative horse-specific kinase gene (*sak*_phi-42e_, GenBank AP019751 [2,118,394..2,119,488:RC]; see below, and Supplemental Files S2b and S2c).

In addition, there were three further prophages. One prophage was located between *rpmF* and *isdB* (encoding 50S ribosomal protein L32 and extracellular haemoglobin-binding protein, cg MLST IDs SAUR1124 and SAUR1125), and another one interrupted the A5IU43=*yfkAB* gene (putative protein/cgMLST SAUR2054). Yet one additional prophage harboured, most notably, the *phi-pdp*-SAU gene (see below). It integrated into the *ebh* gene (cell wall-associated fibronectin-binding protein, in BA000033.2, locus tagMW1324) and carried an integrase gene (VSZG01000002 [4067..5272]) identical to the one in GenBank ARPA01000007.1, which is also associated with a fragment of *ebh*. The prophage integration into *ebh* was observed in both Illumina and ONT sequencing, as well as in an Illumina contig of another CC1 strain, IMT39701 (GenBank VSZF01000024), allowing the assumption that this integrase was site-specific for *ebh*.

IMT39173 carried the beta-lactamase operon, aminoglycoside resistance genes *aacA-aphD* and *aadD*, the tetracycline resistance gene *tet* (L), and the biocide resistance gene *qacA/B* on one contig (Nanopore; GenBank CP176569) or on three contigs (Illumina; VSZG01000030, VSZG01000049, VSZG01000068; Supplemental File S2d), that is, presumably, on a plasmid. The trimethoprim resistance gene *dfrG* was present, and it was as usual accompanied by a gene (GenBank FN433596.1 [500,239..502,191]) encoding an insertion element protein, Q4H3Y2. Both were integrated into the *lukE* gene. With regard to the presence of resistance genes, the isolate appeared to be rather typical, as aminoglycoside, trimethoprim, and tetracycline resistance markers seem to be common among equine *lukP/Q-*positive CC1-MSSA (Supplemental File S2d). Out of the sequences analysed (see above), two *lukP/Q*-positive CC1-MSSA originated from the Berlin/Wernigerode strain collection and four from Austria. In addition, sixteen genomes were published from Texas (Supplemental File S2a). Out of these sequences, thirteen had the tetracycline resistance gene *tet* (L) and the aminoglycoside resistance gene *aadD* on the same contigs, i.e., presumably on the same plasmids (Supplemental File S2d). These thirteen isolates also harboured the biocide resistance gene *qacA/B* and the aminoglycoside resistance gene *aacA-aphD* (which was also present in one of the other sequences), but these genes were localised on different contigs. The beta-lactamase gene *blaZ* was found in seventeen isolates, also on other contigs than the other antimicrobial resistance genes. Two sequences included *dfrA*, four harboured *dfrG*, and eight contained *dfrK*. In strain IMT39701, GenBank VSZF01000041, *dfrG* was also interrupted *lukE* in the same way as described above. If *dfrK* was present, it was located on the same contigs as *tet* (L) and *aadD*. Regarding tetracycline resistance genes, ten genome sequences carried *tet* (L) and one *tet* (K) plus *tet* (L). The chloramphenicol resistance gene *cat*_pC221_ was present once.

### 2.2. CC1-MRSA

No *lukP/Q*-positive CC1-MRSA were identified during this study. The only CC1 *lukP/Q-*positive MRSA is a reference sequence from Japan (JRA307, AP019751; [47]). This sequence did not only include prophages with *lukP/Q* and *scn-eq* (Supplemental Files S3 and S4) as well as a prophage with a putative horse-specific kinase gene, but also a SCC*mec* element with *mecA* and *mecC* [47], the SCC*mec* XI-specific beta-lactamase gene, and two copies of *blaZ*, one as part of a transposon integrated into the strain’s genome and the other one on a plasmid (AP019752).

### 2.3. lukP/Q-Positive CC350-MSSA Isolates and Sequences

Isolate IMT37083 (GenBank: CP176566, VSZD01) belonged, as all CC350 isolates, to *agr* group II and capsule type 5. With an allelic profile of *arcC*-6, *aroE*-79, *glpF*-6, *gmk*-47, *pta*-89, *tpi*-70, and *yqiL*-61, it was assigned to ST1660. It harboured the *egc* enterotoxin gene cluster (comprising *seg*, *seli*, *selm*, *seln*, *selo*, *selu*), one of the lesser known enterotoxin homologues (“*selu2=sel27*”, GenBank NZ_CP019945 [1,942,976..1,943,728]), as well as the haemolysin/leukocidin genes *hlgA/lukF/lukS*, *lukE/D*, and *lukA/B*. Horse-specific virulence factor genes *lukP/Q* and *scn-eq* were present (Supplemental Files S3 and S4). The *cna* gene was present, but *sasG* was absent.

This is in accordance with the other CC350 sequences. These included NCTC5663 (GenBank: LS483317), fifteen sequences from the Berlin/Wernigerode strain collection, and five from Austria, as well as four previously published sequences from the USA and Canada (Supplemental File S2a/b).

With regard to resistance markers, IMT37083 carried *blaZ*, *aacA-aphD*, and *dfrA* on one plasmid (CP176567) while the other German isolates had *blaZ*, *aacA-aphD*, *dfrA*, and *qacC/D* localised on different contigs (Supplemental File S2d). One isolate from the USA (29-017; GenBank: JAAFMT01) carried *dfrG*, *cat* (encoding chloramphenicol resistance, allele as in GenBank X65462.1), and aminoglycoside resistance genes *aacA-aphD*, *aadD*, and *ble*, with the latter two being localised on one contig. The other American, Canadian, and Austrian sequences did not harbour resistance genes. LS483317 included a heavy metal resistance operon with *cadA* (corresponding locus tag SAR0723), *cadC* (SAR0724), and *cadD* (SATW20_00820) as a transposon integrated into the chromosomal *radC* gene (encoding DNA repair protein C; in CP000046.1, locus tag SACOL1707).

### 2.4. lukP/Q-Positive CC816 Isolates and Sequences

The CC816 isolate V353 (GenBank CP176565) originated from a dog, but it was included because of its carriage of *lukP/Q* and of the observation of CC816 in horses (see below and Supplemental File S2a). It belonged to ST816, with an allelic profile of *arcC*-18, *aroE*-140, *glpF*-45, *gmk*-2, *pta-*7, *tpi*-14, and *yqiL*-7. The *spa* type of this isolate was t1294 (04-20-17-111-16-109-24-17). It carried *agr* group II and capsule type 5 alleles but lacked any SCC*mec*-associated markers and any genes associated with antimicrobial resistance. Regarding toxin genes, it carried *tst1* (toxic shock syndrome toxin), *sec*, *sel* (enterotoxin genes C and L), a rare allele of the enterotoxin A gene (*sea*_320E_, [48]; GenBank: AY196686.1 and CP001996.1 [1,144,119..1,144,898]), the *egc* enterotoxin gene cluster and the enterotoxin gene homologue ORF CM14 (GenBank CP010526 [71,533..72,312]). Haemolysin/leukocidin genes *hlgA/lukF/lukS*, *lukE/D*, *lukA/B*, and *lukP/Q* (see below) and *scn-eq* were also present. The genes *lukP/Q* and *scn-eq* as well as *sea*_320E_ were localised on a prophage (Supplemental Files S2 and S3) that had integrated into the lipase gene *lip2=geh* (cgMLST SAUR0317). Another prophage within the A5IT17 gene (encoding a putative protein and including the site where PVL phages integrate in human strains; cgMLST SAUR1313) carried the putative kinase gene *sak*_phi-42e_. In addition, the isolate carried two pathogenicity islands. One included enterotoxin genes *sec* and *sel* as well as *tst1*. The other one, integrated next to *setC=selX* (encoding an enterotoxin-like toxin, in BA000033.2, locus tag MW0345), comprised *scn2*, a gene for a paralogue of staphylococcal complement inhibitor, *vwb3*, a gene for a variant of the “van Willebrand factor” binding protein (GenBank CP084107 [2,030,971..2,031,321:RC] and AM990992 [495,369..496,868], respectively), and D0K398-*xis*, a gene for a putative DNA-binding protein/excisionase (GenBank LS483311 [408,456..408,728]). Regarding CC-associated adhesion factors, it was positive for *cna* but lacked *sasG*.

One Austrian equine isolate as well as five previously published genomes (GenBank JAAFKQ01, JAAFLJ01, JAAFLL01, JAAFMW01, and JAAFNH01 that originated from North American horses) were also assigned to CC816 (Supplemental File S2a). All harboured *lukP/Q* and *scn-eq* as well as *sea*_320E_. One sequence (28-062; GenBank JAAFMW01) apparently lacked *sec/l*, *egc*, and *lukE/D*, which might be due to deletions or to sequencing artefacts. Only one sequence (JAAFLL01) included resistance genes, i.e., *tet* (K) and *cat*_pC221_.

### 2.5. Description of the CC8115 Isolate

Isolate V641 (GenBank CP176564) belonged to ST8115, with an allelic profile of *arcC*-18, *aroE*-1026, *glpF*-427, *gmk*-167, *pta-*109, *tpi*-50, and *yqiL*-2. The first and so far only ST8115 isolate with an entry in the MLST database (https://pubmlst.org/bigsdb?page=profileInfo&db=pubmlst_saureus_seqdef&scheme_id=1&profile_id=8115, as accessed on 20 November 2024) originated from the milk of a mastitic animal of undisclosed species affiliation and was isolated in 2021 in Xinjiang, People’s Republic of China. The *spa* type of isolate V641 was t14869 (04-82-17-25-16-16-16-17), a type found just once in Kenya (https://spa.ridom.de/spa-t14869.shtml, as accessed on 20 November 2024). We were not able to identify further published genomes of this clonal complex/sequence type for comparison.

V641 belonged to *agr* group IV and capsule type 5. It lacked any resistance genes and SCC*mec* markers, although it carried two putative DNA helicase genes (corresponding to GenBank CP024649 [51,055..51,900] and [51,897..53,161]) in the position of a SCC element. The *egc* enterotoxin gene locus was present; *tst1*, *sec*, and *sel* were absent. The enterotoxin A allele *sea*_320E_ was present, as well as the *lukP/Q* genes and *scn-eq*. Other haemolysin/leukocidin genes *hlgA/lukF/lukS*, *lukE/D*, and *lukA/B* were observed. The genes *cna* and *sasG* were absent.

The isolate carried three prophages. One integrated into the haemolysin beta gene *hlb*. This prophage comprised the virulence-associated gene *virE* (JRA307, GenBank AP019751 [2,145,911..2,148,358:RC]) and the putative kinase gene *sak*_phi-42e_. A second prophage, integrating into the lipase gene *lip2=geh*, was the prophage that carried *lukP/Q*, *sea*_320E_, and *scn-eq* (Supplemental Files S2 and S3). A third prophage was located directly downstream of the *sufB* gene (for iron-sulphur cluster assembly protein B, cgMLST ID SAUR0895), but it did not contain any of the markers discussed herein. Furthermore, the isolate harboured a pathogenicity island (different than the one described for V353) with *scn2*, *vwb3*, an integrase gene, and AIO21657*-xis*, a putative phage excisionase (GenBank AJ938182 [2,035,765..2,036,082:RC]).

### 2.6. Phages/Prophages Carrying lukP/Q

Phages carrying *lukP/Q* from the study strains as well as the original *lukP/Q* phage (GenBank LT671578) and the *lukP/Q* prophages from the CC1-MRSA strain JRA307 (GenBank AP019751.1) and the CC350-MSSA strain NCTC5663 (GenBank LS483317.1) were analysed in detail. Besides, *lukP/Q* genes were detected in 71 isolates or genome sequences that belonged to CC1, CC6, CC97, CC133, CC350, CC398, CC816, ST2679, and ST6242.

All these phages shared the same variant of a site-specific integrase, the presence of *lukP/Q*, and a gene annotated as an equine staphylococcal complement inhibitor (*scn-eq*; GenBank LT671578.1 [3266..3610]). All phages are integrated into the *lip2=geh* gene. In the sequence of the original *lukP/Q* phage (GenBank LT671578), remnants of this gene as well as of neighbouring genes for A5IPQ2 (putative hydrolase gene, cgMLST SAUR0318) and Q6GJZ4=*namA* (oxidase family protein, cgMLST SAUR0319 (SAR_RS01595)) could be identified, which confirmed the identification of this integration site. In 68 of the 71 *lukP/Q-*positive sequences, at least parts of the *lip2=geh* gene were detected on the same contigs as *lukP/Q*. In addition, all 71 sequences carried the *lip2=geh-*associated integrase gene (as represented by SACOL0318). Thus, it can be assumed that the vast majority, possibly even all, *lukP/Q*-phages integrate into that gene. All genome sequences that harboured *lukP/Q* also carried *scn-eq*. In all genomes but one (VSYV01), it was found on the same contig as at least one of the two *lukP/Q* genes, allowing the assumption that it generally is located on the same phage as the horse-specific leucocidin genes.

The *lukP/Q* phage/prophage sequences can roughly be divided into three groups or families, based on gene content, with phages from one group being similar but not identical to each other (see Table 1/Supplemental Files S2b and S2). We tried to identify phages by mapping marker genes (as indicated in Table 1/Supplemental File S3) on the contigs of published genome sequences in order to assign *lukP/Q-*phages to the three groups described above. Due to the fragmentation of the sequences in multiple contigs and the simultaneous presence of several prophages in the same *S. aureus* genome, this was not in all cases perfectly and unambiguously possible, but the following picture emerges (Table 1, Supplemental Files S2b and S3).

The first group comprised *lukP/Q* phages from IMT39173 (see GenBank VSZG01 as fragmented Illumina sequence and GenBank CP176568/Supplemental File S3 for a contiguous sequence) and JRA307 (GenBank AP019751). Another fourteen CC1 sequences harboured “Group 1” prophages, and one CC1 prophage could not unambiguously be assigned to any group. The only other CC in which “Group 1” prophages were identified was CC97 (see Supplemental File S2b).

The second group of *lukP/Q* phages is represented by IMT37083 (GenBank CP176566, VSZD01, CC350), by the phage from the CC133 strain 3711 ([31], GenBank LT671578) in which *lukP/Q* was first described, and by strain NCTC5663 (GenBank LS483317, CC350). All CC133 (*n* = 11) and CC350 (*n* = 27) sequences that harboured *lukP/Q* carried markers in accordance with the presence of “Group 2” prophages. This included German and Austrian isolates as well as two CC350 sequences from the US and two from Canada, as well as the Swedish strain NCTC5663. For one Texan CC398-MSSA, a “Group 2” prophage was identified, with all relevant markers being localised on one contig (strain 59-071, JAAFLK010000001.1), where it is also integrated into *lip2=geh*. In four German CC398-MRSA, a prophage of this group can be suspected but not proven because the relevant genes were split across several contigs and because of the presence of other marker genes on the same contigs as “Group 2” markers. Two published CC6 sequences harboured most likely “Group 2” prophages. Seven of the CC1 sequences carried a “Group 2” prophage. Similarly, CC97 sequences had either “Group 1” or “Group 2” prophages. The two sequences of ST2679 and ST6242 (SAMN17368090, SAMN17368091; [37]) are very similar; they might be merged together as CC873 (MLST: 6-79/384-113-83-7/759-114-102) and they apparently also harbour a “Group 2” prophage.

The third group was identified in the CC816 and CC8115 isolates (V353 and V641, GenBank CP176564 and CP176565) described herein. Most conspicuously, they carried not only *lukP/Q* but also the distinct enterotoxin A allele *sea*_320E_. All five published CC816 sequences (GenBank JAAFKQ01, JAAFLJ01, JAAFLL01, JAAFMW01 and JAAFNH01) as well as one Austrian isolate also harboured *sea*_320E_, and this, together with the presence of other marker genes, indicated that they carried “Group 3” prophages as observed in the study isolate V353. Isolate V641 had also a prophage belonging to this group.

Because a study isolate of CC816 originated from a dog, published genomes from dog isolates also were screened for the presence of *lukP/Q* and *scn-eq* (*n* = 108, see www.bv-brc.org/view/Taxonomy/1279#view_tab=genomes&filter=and(eq(genome_quality,%22Good%22),eq(host_common_name,%22Dog%22),keyword(aureus)) as accessed on 17 January 2024). This yielded not a single *lukP/Q*- or *scn-eq*-positive case. In addition, 95 genomes of *S. aureus* from sheep and goats were searched (www.bv-brc.org/view/Taxonomy/1279#view_tab=genomes&filter=and(eq(genome_quality,%22Good%22),or(eq(host_common_name,%22Goat%22),eq(host_common_name,%22Sheep%22)),keyword(aureus)); duplicates and *S. xylosus* removed, accessed 2 February 2024). While 33 yielded hits for *lukM* and *lukF-P83*, 21 genome sequences harboured *lukP*, an observation explained further below (Section 2.8).

### 2.7. Equine Strains/Sequences That Did Not Contain lukP/Q Genes

A single genome sequence of an equine CC1, one CC133, and several CC398 lacked *lukP/Q* genes (Supplemental File S2b). None of the published genomes of equine CC5, CC8, CC22, CC45, CC188, and CC4215 isolates harboured *lukP/Q*, supporting the notion that these were originally human strains secondarily transmitted to horses. Instead, in some of the previously published CC8 sequences, *lukS/F*-PV genes were identified (Supplemental File S2a). All these sequences were assigned to the USA300, which is also consistent with a human-to-horse transmission.

### 2.8. Strains and Prophages with the Chimeric Phage-Borne Leukocidin

A putatively chimeric leukocidin was identified in a CC522-MSSA strain, 17CS1042 from a Thuringian goat, recently sequenced by the authors, GenBank CP138360.1. Its prophage sequence is also provided in the Supplemental Files S2 and S4. The same leukocidin was noted in an older sequence, a CC130-MSSA, contig 64 of isolate SA-006, GenBank JXHY01000064 from Switzerland, published in 2015 [49] and in an entry from the PubMLST database (ST522; https://pubmlst.org/bigsdb?db=pubmlst_saureus_isolates&page=seqbin&isolate_id=24359, retrieved at 8 November 2024).

**Figure 1:**
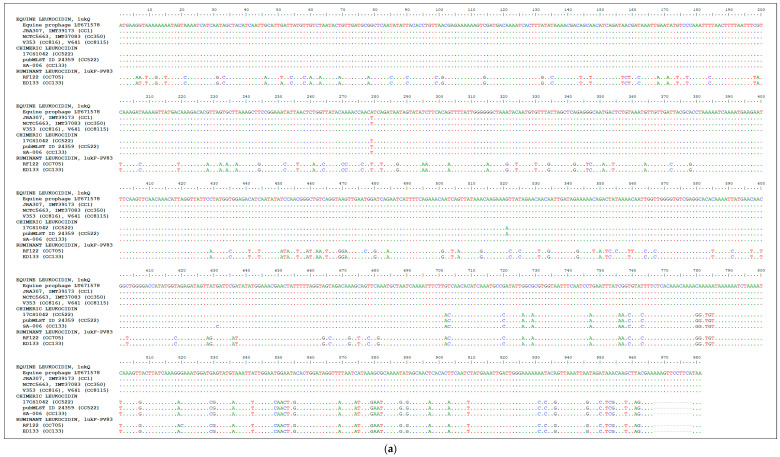

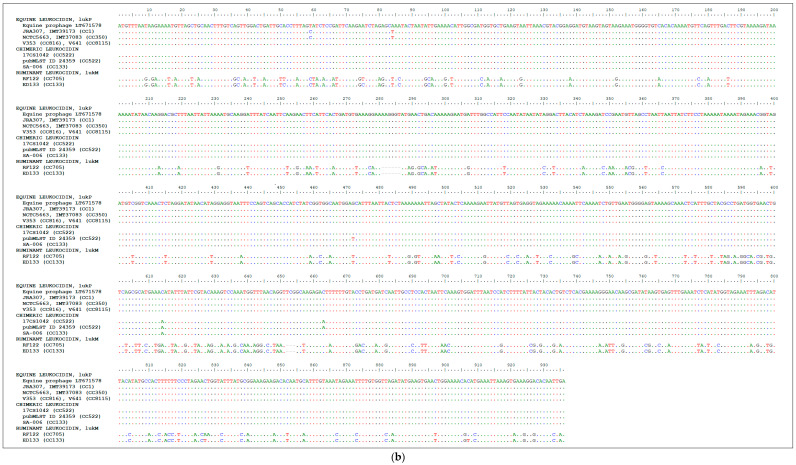
(**a**) Alignment of *lukQ* and *lukF-P83*, showing that sequences from CP138360, pubMLST ID 24359, and JXHY01000064 match the sequence of *lukQ* until position 702 and the one of *lukF*-PV83 henceforth. (**b**) Alignment of *lukP/lukM* sequences, showing that the sequences from CP138360, pubMLST ID 24359, and JXHY01000064 match the sequence of equine *lukP* rather than the one of bovine *lukM*.

The genes encoding the *lukF* component in CP138360 and JXHY01000064 (reverse complement, RC) [20,970..21,938] were nearly identical to each other (Figure 1a). In positions 1 to 701, they differed in one (nucleotide and deduced amino acid) position from each other and from the *lukQ* sequences (and shared an additional silent SNP with some, but not all, *lukQ* sequences). In positions 702 to 969, they were identical to *lukF-P83* from the CC133 strain ED133 (GenBank CP001996.1 [1,975,486..1,976,454]) and differed in a single non-synonymous SNP from the one of the CC705 strain RF122 (GenBank AJ938182.1 [851,630..852,598]). The *lukF* component of pubMLST ID 24359 (ERR525099_A131030_NODE_133, [15,045..16,013]) was identical to the one in CP138360.

The genes encoding the *lukS* component in CP138360 and JXHY01000064 RC [21,940..22,875] differed from each other in one nucleotide and deduced amino acid position. They diverged from the *lukP* sequences (Figure 1b) in another non-synonymous SNP. In addition, two further SNPs (one silent, one not) were observed, affecting some but not all *lukP* sequences. The *lukS* component of pubMLST ID 24359 (ERR525099_A131030_NODE_133 [16,015..16,950]) was, except for one SNP, also identical to the one in CP138360.

In isolate 17CS1042, the prophage carrying this chimeric leukocidin was integrated at a different site as the *lukP/Q* phages in horse strains, i.e., not into the *lip2=geh* gene, but between genes encoding Q5HEP8 (for a putative protein, cgMLST SAUR2053) and A5IU44 (for an acyl-CoA thioesterase, cgMLST SAUR2055), disrupting A5IU43 (putative protein, cgMLST ID SAUR2054). This is the same position as the *lukM/lukF-P83* phage in ED133 (GenBank CP001996) holds (Supplemental File S1). Accordingly, this prophage also harboured another integrase gene than the *lukP/Q* prophages, which is nearly identical to the corresponding one in ED133 (CP001996 [2,017,417..2,018,463]; with 8 SNPs on a length of 1046 bp).

For the leukocidin prophages in the other two sequences, SA-006 and pubMLST ID 24359, the same integration site also safely might be assumed as Q5HEP8 and a fragment of A5IU43 are in both cases localised on the same contigs as the chimeric leukocidin (JXHY01000064 RC, [18,152..18,313] and [18,537..18,591]; ERR525099_A131030_NODE_133, [11,021..11,182] and [11,406..11,460], respectively).

Although it was present on all *lukP/Q* phages, the *scn-eq* gene was not identified on any of the sequences of phages with a chimeric phage-borne leukocidin.

### 2.9. Phages/Prophages Carrying the Putative Kinase Gene

An interesting observation concerned a putative kinase gene. During automated annotation, “staphylokinase” was identified based on sequence identity to a gene in NCTC5663 (locus tag NCTC5663_00926; LS483317 [935,657..936,751]). However, the presence of staphylokinase (in MW2, locus tag MW1885; BA000033 [2,049,256..2,049,747:RC]) was ruled out based on array hybridisations of the strains in question. Besides, the staphylokinase gene has a length of 492 bp, while the one from phage 42e encompasses 1095 bp. Currently (as of June 2024), there are three annotated entries of that sequence in GenBank (in AY954955, AP019751, and LS483317), in which it is once annotated as staphylokinase, once as kinase, and once as “putative protein”. Thus, it might be assumed that this gene represents a yet unknown kinase gene, preliminary dubbed *sak*_phi-42e_ herein. It is phage-borne, albeit not located on the *lukP/Q* phages, and it is present in a considerable number of horse isolates (see below and Supplemental File S2c).

The putative kinase gene was found in the four strains scrutinised herein, and it is also present in JRA307 (GenBank AP019751, CC1) and NCTC5663 (GenBank LS483317, CC350), as well as, of course, in phage 42e (GenBank AY954955). Details are provided in Supplemental File S5. It was also detected in 59 of the other genome sequences and isolates analysed that all belonged to CC1, CC97, CC133, CC350, or CC816 (Supplemental File S2c). However, it was not found in all sequences belonging to these respective CCs, which still in some cases might be an artefact due to fragmentation of the sequences.

It was absent from all equine CC5, CC6, CC8, CC22, CC45, CC188, CC398, CC873, and CC4215 sequences analysed. It was neither identified in the 108 genome sequences of dog strains nor in the 95 sheep and goat strains. There was also not a single *lukP/Q*-negative strain positive for *sak*_phi-42e_.

The prophages carrying *sak*_phi-42e_ integrated into different positions of the staphylococcal genome. The allelic variants of the phage integrase genes were found to be in accordance with the integration site of the respective prophages, with an MW1442-like integrase being associated with A5IT17, an MW1939-like integrase with *hlb*, and a SARLGA251_07760-like one being situated next to *sufB*. In the CC1 strains JRA307, GenBank AP019751.1, and IMT39173, as well as in the ST8115 isolate V641, the *sak*_phi-42e_ prophage was integrated into the haemolysin beta gene, as phages carrying the staphylokinase gene *sak* in human strains also do. This might also be the integration site for phage 42E, according to the identity of its integrase gene. In the CC350 strains IMT37083 and NCTC5663, GenBank LS483317.1, prophages carrying *sak*_phi-42e_ were localised between *sufB* and Q2YWM5 (cgMLST IDs: SAUR0895 (SAR_RS04475) and SAUR0897 (SAR_RS04485)). In the ST816 isolate V353, this prophage is integrated at the *graD/*A5IT17 site (within cgMLST SAUR1313), i.e., the site where human strains carry PVL prophages.

Unfortunately, the genes associated with the kinase prophage were in most short-read sequences scattered across several contigs. In the Illumina sequence of IMT39173, GenBank VSZG01, this prophage was fragmented in as much as twelve contigs, as shown in a direct comparison with a Nanopore sequence of the strain’s genome (see Supplemental File S3). Since most *S. aureus* isolates contain several prophages (as much as five in IMT39173), it is not clear which fragments originated from which prophage. Thus, their kinase prophages cannot be described in detail, and they cannot unambiguously be categorised. Based on the long-read sequences (Supplemental File S5), the *hlb-*integrating kinase prophages/phages are very similar to each other, with phage 42e (GenBank AY954955) being the most divergent one of them. The A5IT17-integrating prophage in V353 (CC816) also resembles them, except for the integrase gene and the following ca. 6500 bp, which are dissimilar. This might be attributed to a recombination event between different phages/prophages.

### 2.10. The Gene Encoding a Phage Defence Protein of S. aureus

An additional, noteworthy marker found on the kinase-carrying prophage of the CC1 strain JRA307 was a gene (*phi-pdp*_SAU, GenBank AP019751.1 [2,117,106..2,117,942]) encoding the phage defence protein of *S. aureus* that causes abortive infection by Kayviruses, thus providing immunity of *S. aureus* populations against these lytic phages [50].

While the kinase-carrying phage from IMT39173 was similar to the corresponding prophage from JRA307, the position of this gene was occupied by three other genes (ST42eORF161, ST42eORF048, ST42eORF037; NC_007052.1 [24,767..25,670]) that also could be found in phage 42e and in the ST8115 isolate V641, indicating a possible recombination affecting *hlb-*integrating kinase-phages (Supplemental File S5). Nevertheless, the *phi-pdp*-SAU gene was present in IMT39173, albeit localised on another phage.

In addition to JRA07 and IMT39173, the *phi-pdp*_SAU gene was identified (Supplemental File S2d) in 25 genome sequences, including eleven further CC1 sequences, six CC97, three CC350, and five CC398 sequences. It was usually co-localised with *sak*_phi-42e_, being found on the same contigs of fragmented Illumina sequences (i.e., in seven CC1, six CC97, and three CC350 sequences, Supplemental File S2c). The five CC398 strains did not harbour *sak*_phi-42e_; here *phi-pdp*_SAU was co-localised with *virE*.

### 2.11. Morphology and Nanopore Sequencing of Phages Induced from the Study Strains

All bacteriophages exorcised from strains V353, 17CS1042, IMT37083, and IMT39173 had morphological characteristics of icosahedral, mildly elongated or prolate heads, thin, non-contractile tails, and distinct baseplates (Table 2).

The isolated phage DNA was Nanopore-sequenced in parallel to the TEM investigations. For the five strains, one to three prophages each were identified by sequencing (Supplemental File S3).

#### 2.11.1. Phages from the CC1 Isolate IMT39173

**Figure 2:**
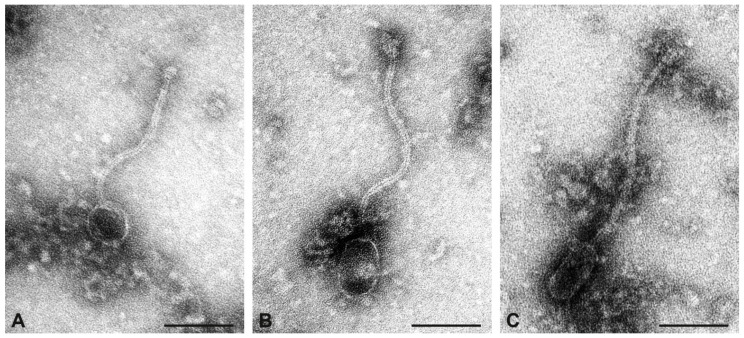
Transmission electron micrographs of phage particles from the preparation of strain IMT39173. (**A**), icosahedral phage. (**B**), prolate phage with large head diameter and oval shape. (**C**), prolate phage with small head diameter and angular shape. Negative contrast preparation with uranyl acetate. Size bars = 100 nm.

The preparation of the CC1 isolate IMT39173 contained numerous phages. Three different morphotypes could be distinguished: icosahedral phages, prolate phages with a large head diameter and oval shape (prolate-thick), and prolate phages with a small head and angular shape (prolate-thin, Figure 2). The dimensions of thick and thin prolate phages were comparable to those from strain V353. Icosahedral phages and thick prolate phages were present with comparable frequency, while thin prolate phages were rarely encountered. Sequencing identified three phages. One, at a very high coverage (=1884) carried *lukP/Q*. Another one carried the putative kinase gene *sak*_phi-42e_ (coverage = 1580). In addition, there was a third phage that included a SAOV_1070c-like integrase gene, which thus can be expected to have integrated between *rpmF* and *isdB* (encoding 50S ribosomal protein L32 and an iron-transport associated protein, SAUR1124, and SAUR1125).

#### 2.11.2. Phages from the CC350 Isolate IMT37083

**Figure 3:**
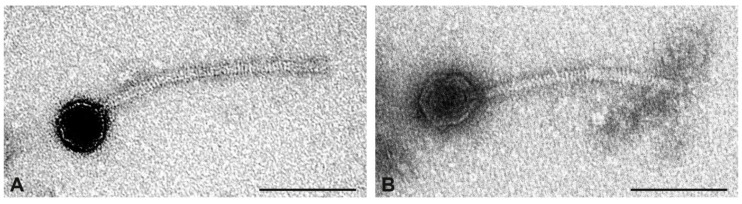
Transmission electron micrographs of phage particles from the preparation of strain IMT37083. (**A**), icosahedral phage. (**B**), phage with mildly elongated head. Negative contrast preparation with uranyl acetate. Size bars = 100 nm.

Many phages were present in the preparation of CC350 isolate IMT37083. Phage particles had similar head diameters but differed in head length (Figure 3). In addition to icosahedral phages, phage particles with mildly elongated heads (5–11 nm more than head diameter) were seen. Icosahedral phages were more frequent than phages with elongated heads. Sequencing of the phage preparation also revealed two phages: one with high coverage (=664) carrying *lukP/Q* and one at low coverage (=22) that harboured *sak*_phi-42e_.

#### 2.11.3. Phages from the CC816 Isolate V353

**Figure 4:**
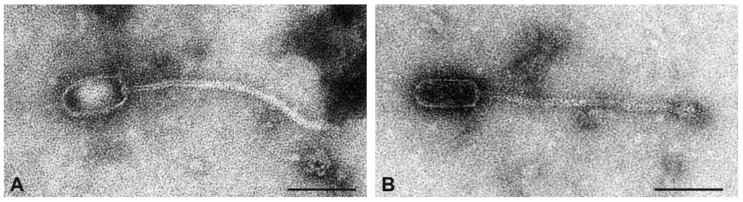
Transmission electron micrographs of phage particles from the preparation of strain V353. (**A**), prolate phage with large head diameter and oval shape. (**B**), prolate phage with small head diameter and angular shape. Negative contrast preparation with uranyl acetate. Size bars = 100 nm.

Many phages were seen in the preparation of the CC816 isolate V353. All particles had prolate heads. Two types of phages could be distinguished based on the diameter and shape of heads: phages with a large head diameter and oval shape (“prolate-thick”) and phages with a small head diameter and angular shape (“prolate-thin”, Figure 4). There were also differences in the length of tails. Both types of phages were present in approximately equal numbers. Sequencing of the phage preparation, however, yielded only one phage at a low coverage of 56, and this was the phage carrying *sak*_phi-42e_.

#### 2.11.4. Phages from the CC8115 Isolate V641

**Figure 5:**
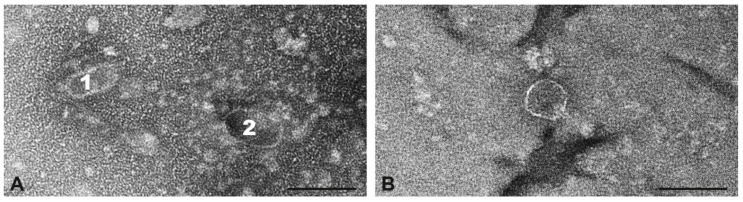
Transmission electron micrograph of incomplete phage particles from the preparation of strain V641. (**A**), two prolate phage particles, one with small head diameter and angular shape (1) and one with large head diameter and oval shape (2). (**B**), head of an icosahedral phage particle. Negative contrast preparation with uranyl acetate. Size bars = 100 nm.

In the preparation of isolate V641, no complete phage particles were detectable. Based on size and shape of isolated heads, prolate phages with an oval shape and a diameter of 50–56 nm, prolate phages with an angular shape and a diameter of 35–45 nm, and icosahedral phages with diameters from 50 nm to 60 nm could be distinguished (Figure 5). Sequencing of the phage preparation identified only one phage at low coverage (=79), which was identified as the *sufB*-integrating phage known from the strain’s genome that carried neither *lukP/Q* nor *sak*_phi-42e_.

#### 2.11.5. Phages from the CC522 Isolate 17CS1042

**Figure 6:**
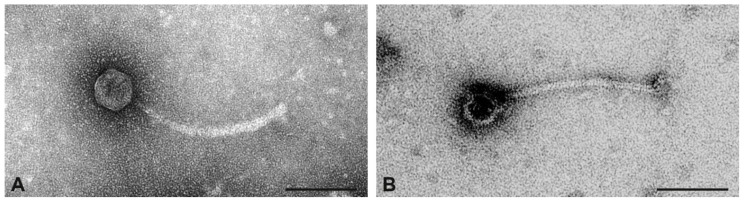
Transmission electron micrographs of phage particles from the preparation of strain 17CS1042. (**A**), large icosahedral phage. (**B**), small icosahedral phage. Negative contrast preparation with uranyl acetate. Size bars = 100 nm.

A moderate number of phages was observed in the preparation of isolate 17CS1042. All particles had icosahedral heads. Two types of phages could be discriminated based on the diameter of heads: large icosahedral phages with an average diameter of 54 nm and small icosahedral phages with an average diameter of 48 nm (Figure 6). Tails and baseplates were similar. The larger phages were more frequent than the smaller phages.

Sequencing this preparation showed one phage with moderate coverage (=137) that carried the chimeric leukocidin and one additional phage (coverage = 78), which carries a SAOV_1070c-like integrase gene and is integrated between *rpmF* and *isdB* (CP138360.1 [1,103,911..1,147,648]).

## 3. Discussion

All phages observed herein could be assigned to Siphoviruses. Phages/prophages that harbour *lukP/Q* and *scn-eq* could be roughly divided into three groups or clades. They uniformly carried both *lukP/Q* and *scn-eq*, as well as the integrase gene associated with integration into *lip2=geh*.

“Group 1” phages were observed in some, but not all, CC1 and CC97 sequences. “Group 2” prophages were distributed across the highest number of *S. aureus* CCs, being identified primarily in CC133 and CC350 but also in CC6, CC398 and CC873 (see above), as well as in some other CC1 and CC97, while “Group 3” was associated with the poorly known clonal complex CC816 and the single isolate of CC8115. The ST350 strain NCTC5663 (GenBank LS483317, CC350) has originally been isolated in 1933 in Sweden (https://www.ccug.se/strain?id=2353, accessed on 27 November 2024). Thus, it represents one of the oldest *S. aureus* genomes currently available. This indicates that *lukP/Q*, *scn-eq*, and *sak*_phi-42e_ did not recently emerge but places their emergence at least close to a century into the past. The observation that “Group 2” phages apparently have a wider and more diverse range of staphylococcal hosts might, for that reason, simply be explained by assuming that they are the most ancient lineage of *lukP/Q* phages/prophages, having had more time to spread across and to adapt to staphylococcal populations of diverse clonal backgrounds.

Phages/prophages that carry *sak*_phi-42e_ differed in their integration sites and in the carriage of site-specific integrase genes. There were A5IT17/*graD*-, *hlb*-, and *sufB*-integrating prophages (Supplemental File S1). A further classification of these prophages was currently not possible due to fragmentation of short-read genomes into multiple contigs and the concurrent presence of other prophages that made it impossible to assign a given contig to a particular prophage.

The strains sequenced herein as well as reviewed sequences and literature identified three phage-transmitted virulence factors of interest that might be associated with pathogenesis in horses. These are *lukP/Q* and *scn-eq*, as well as a putative kinase gene provisionally tagged *sak*_phi-42e_.

For the former two, there are not only epidemiological associations but experimental evidence showing how they interact with the equine immune defences [31,32]. For the latter one, *sak*_phi-42e_, virtually nothing is known beyond a computerised classification as kinase gene. However, data shown here indicate it to be strongly associated with phages/prophages from horse isolates of *S. aureus*, and this should prompt further investigations on a possible role in pathogenesis in horses.

Another interesting issue is recombination between phages/prophages. Some of the sequences discussed might provide evidence for such recombination events. Although the kinase prophages from IMT39173 and JRA307 were very similar to each other, the gene encoding the phage defence protein in the former was replaced by three other genes that were also observed in phage 42e and in the kinase phage of ST8115 isolate V641. However, the *phi-pdp*-SAU gene was still present in IMT39173, but on another phage. Another case of recombination might have resulted in a change of the integration site of the kinase phage in the CC816 isolate V353, where a region of about 6500 bp including the A5IT17-specific phage integrase differs from the otherwise similar *hlb-*integrating kinase phages/prophages of other sequences. Given that many genes of the latter also resemble genes from the A5IT17-specific PVL prophage of MW2, it is probable that the *hlb-*integrating kinase phages are the recombinant ones that secondarily acquired the *hlb*-specific recombinase and its neighbouring genes.

Finally, the “chimeric” *lukF* component of the leukocidin in CC522 might most likely be a product of a recombination event involving phages carrying *lukQ* and *lukF-*PV83. In general, temperate phages of *S. aureus*, including those belonging to *Siphoviridae*, are known to undergo frequent recombination events [51,52,53]. This leads to mosaic genome structures where different segments have distinct evolutionary histories. *S. aureus* phage genomes typically have a modular organisation, which facilitates the exchange of functional units between phages through recombination. Recombination can occur when multiple phages co-infect the same bacterial cell. The prevalence of prophages in *S. aureus* genomes thus provides ample opportunity for genetic exchanges between phages. After all, one of the study strains (IMT39173) carried as much as five different prophages.

Because one study isolate of CC816 originated from a dog, published genomes of dog isolates were also screened for the presence of *lukP/Q* and *scn-eq*. As mentioned above, this yielded not a single positive case, so we still assume that our isolate represented a “horse-specific” lineage that just accidentally was transmitted to a dog. However, a detection of *lukP/Q*+*scn-eq-*positive strains might be in accordance with the previously noted low host specifity of these virulence factors [31,32]. Despite the unresolved role of these genes in dog isolates, the issue of *lukP/Q* emphasises the role, bacteriophages play in the determination of the host specificity of *S. aureus*. CC1 is a widespread clonal complex. A high percentage of human isolates carries *lukS/F*-PV phages (encoding PVL), which are integrated at the A5IT17/*graD* site, while horse isolates carry *lukP/Q*. Similarly, natively human (East Asian clade) CC398 isolates are frequently PVL-positive. Livestock-associated (European clade) CC398 isolates lack these genes, while horse-associated CC398 isolates, at least occasionally, are positive for *lukP/Q*. One might speculate that they secondarily acquired these genes from native equine lineages during adaptation to horses as a new host, and indeed, *lukP/Q* prophages from CC398 are closely related to those from CC350, which represent, as discussed above, the oldest and most widely spread group of *lukP/Q* prophages.

Interestingly, all *lukP/Q* prophages, regardless of group affiliation, integrate into the *lip2=geh* gene ([54], Supplemental File S1). Because of this integration, strains are expected to lose the ability to produce the Lip2 lipase, which appears to be important for skin colonisation and fatty acid detoxification [54]. This might explain why *lip2=geh* disruption is, otherwise, relatively rare and why its frequency varies depending on the isolation site of the bacteria [54]. The loss of Lip2 function may be more tolerable not only in certain environments (like blood or nose) but also in non-human, that is, equine, hosts where its lipase activity might be less critical for bacterial survival and/or where the acquisition of *lukP/Q* might compensate for the loss of the *lip2=geh* gene’s function. A similar trade-off occurs in many human strains of *S. aureus* in which a prophage insertion into the *hlb* gene compensates the loss of its function by introducing the *scn/chp/sak/sae* immune evasion cluster [55,56], while in ruminants, the sphingomyelinase activity of the *hlb* gene product appears to be more crucial than the presence of this cluster [57].

The enterotoxin gene *sea*_320E_, observed here in CC816 and CC8115, has also previously been found in horse isolates as well as in other animal isolates of *S. aureus*. It is obviously not mandatorily linked to *lukP/Q*. In the genome of the “small ruminant” strain ED133 (CC133; GenBank CP001996.1, locus tag SAOV_1120), it is situated on a prophage integrated between *isdB* and *rpmF*. In the sequence of ILRI_Eymole1/1 from a Kenyan dromedary (CC30, GenBank LN626917.1 [888,297..888,794]), it is associated with a prophage fragment situated at the A5IT17 integration site, but both, the prophage and the enterotoxin gene, are truncated by a transposon integration. With regard to localisation, it thus appears to differ from other *sea* alleles that are in human strains localised on *hlb*-converting prophages.

Of note, the ancient NCTC5663 strain (isolated in 1933; see above) lacks all resistance genes common in other equine isolates, which is not surprising as none of these substances was then in use. However, it carries a cadmium resistance operon (as a transposon, integrated into *radC)*. The *radC* gene is associated with DNA repair mechanisms but frequently serves as an integration site for various transposons [58,59,60]. The cadmium resistance operon in question confers resistance not only to cadmium but also to zinc, as discussed in [61] referring to *cadA/C*-positive ATCC^®^12600=DSM 20231 (GenBank: AMYL01). Thus, one might speculate that zinc-containing ointments for the treatment of dermatitis and minor skin injuries in horses were already then in usage as they still are today (CC1-MRSA JRA307, AP019751.1, also carries that operon but as part of its SCC*mec* element).

A considerable number of strains and genome sequences from more recent times contain trimethoprim resistance genes (*dfrA*, *dfrG*, *dfrK*). The high rate of *dfr*-genes indicates a common usage of trimethoprim (in combination with sulfamethoxazole, as co-trimoxazole) in horses posing a significant selective pressure. Intriguingly, the above-mentioned CC8/ST612-MRSA-IV strain that spread from humans in South Africa to horses in Australia was also resistant to trimethoprim, mediated by carriage of *dfrA* on a plasmid and selected for by the common usage of co-trimoxazole in Southern Africa for prophylaxis and therapy of AIDS-associated *Pneumocystis jirovecii* pneumonia [35].

The observation that *dfrG* integrated into *lukE* in two CC1 sequences might suggest that i) these isolated might be clonally related (although no direct connection could be established: they originated from one site but were sampled three months apart from unrelated animals), and ii) that the evolutionary advantage provided by the presence of *dfrG-*associated trimethoprim resistance might outweigh the disadvantage caused by the loss of the function of *lukE*, or that the latter might altogether be expendable due to a presence of *lukP/Q*.

The evolution of antimicrobial resistance in *S. aureus* strains isolated from horses thus reflects the history of use of anti-infective compounds in equine medicine. From early resistance to metals like cadmium and zinc to the widespread presence of trimethoprim resistance genes in modern isolates, these genetic adaptations highlight the selective pressures exerted by common veterinary treatments and underscore the importance of judicious antimicrobial use in both animal and human medicine.

## 4. Conclusions

*S. aureus* is a versatile pathogen being able to infect, or to colonise different host species. Its host specificity is at least in part determined by the presence of prophages. Although recent years witnessed a transmission of human- and livestock-associated MRSA into horses, equine strains of *S. aureus* usually carry prophages with specific leukocidin genes, a putative kinase gene, and a complement inhibitor. These prophages can be found across several clonal complexes, and they have been extant for more than ninety years.

## 5. Materials and Methods

### 5.1. Isolates and Sequences

Four isolates were for this study sequenced using Oxford Nanopore Technology (ONT). This included one ST1- and one ST1660-MSSA from diagnostic horse samples that were known as *lukP/Q*-positive based on previous Illumina sequencing (accession numbers VSZD00000000 and VSZG00000000). The ST1 isolate IMT39173 originated from the inflamed synovium of a five-year-old English Thoroughbred gelding that was hospitalised and finally euthanised because of septic arthritis. The CC350 isolate IMT37083 was cultured from a swab of an exsudating wound of a 14-year-old Shetland Pony mare that eventually recovered.

Furthermore, two isolates, ST816 and ST8115, were included from diagnostic samples that were identified by microarray hybridisation (see below) as representative of rare and poorly known lineages prompting genome sequencing, which in turn revealed the presence of *lukP/Q* prophages. The ST816 isolate V353 originated from a wound swab of a two-year-old dog. It was included because of the presence of *lukP/Q* and because CC816 belongs to the lineages prevalent in horses. The ST8115 isolate V641 was cultured from a skin swab from a horse.

The genome sequences of these four isolates are submitted to GenBank, accession numbers CP176564-CP176569.

A sequence of a ST522 strain from a nasal swab of a healthy Thuringian goat (NZ_CP138360.1) was included for comparison because its sequence obtained for an earlier project [62] revealed a prophage with leukocidin genes reminiscent of *lukP/Q*.

In addition to the genomes mentioned above, 42 short-read (Illumina) genome sequences were analysed that were obtained by the Berlin/Wernigerode groups from diagnostic horse samples. These included two isolates assigned to *lukP/Q*-positive CC1-MSSA, fifteen *lukP/Q-*positive CC350 (ST1660)-MSSA as well as four *lukP/Q*-positive CC398-MRSA. Twenty-one CC398-MSSA from that collection proved to be *lukP/Q*-negative. Their BioProject accession numbers are PRJNA435710, PRJNA449454, PRJNA473644, and PRJNA561466. For the GenBank accession numbers of these isolates, see Supplemental File S2a.

Finally, eleven short read (Illumina) genome sequences of *lukP/Q*-positive isolates from horse samples were provided by the VetMed/AGES groups (Vienna, Austria). Five of them were CC350-MSSA, four CC1-MSSA and one each belonged to CC133 and CC816, respectively. Details are listed in Supplemental File S2a; the BioProject accession number is PRJNA1194107.

### 5.2. Previously Published Sequences Analysed for Comparison

In addition, two fully assembled and annotated genomes from GenBank were analysed. These included one equine CC1-MRSA from Japan (JRA307, AP019751, see [47]) and a CC350-MSSA strain NCTC5663 (GenBank: LS483317, which is *lukP/Q*-positive but for which no host was provided in the GenBank record). Furthermore, published (short-read) sequences of another 101 isolates were analysed for which “horse”/”*Equus caballus*” was provided as host species (https://www.bv-brc.org/view/Taxonomy/1279#view_tab=genomes&filter=and(eq(host_common_name,%22Horse%22),eq(genome_quality,%22Good%22),keyword(aureus)); for the accession numbers, see Supplemental File S2a).

Finally, two short-read sequences of equine MSSA isolates were included (SAMN17368090, SAMN17368091) because they belonged to novel sequence types ST2679 and ST6242 [37] that were neither represented by nor related to any of the other sequences discussed herein.

These sequences of the original *lukP/Q* phage of strain 3711 (CC133, from a horse from the UK; GenBank LT671578; [31]) and of phage 42e (GenBank AY954955; [63]) were also analysed.

### 5.3. Microarray-Based Typing

A preliminary characterisation of isolates, including the detection of resistance genes, virulence markers, and other genes of interest, as well as an assignment, was performed using a microarray-based assay (INTER-ARRAY, Bad Langensalza, Germany). The microarrays, related protocols and methods, as well as probe and primer sequences, have previously been described in detail [7,64,65].

### 5.4. Illumina Sequencing

Isolates collected by the Berlin/Wernigerode and Vienna groups (Supplemental File S2a) were subjected to whole genome sequencing by Illumina technology as described previously [62,66].

In short, from Austrian isolates, DNA was isolated using the MagAttract HMW DNA Kit (Qiagen, Hilden, Germany). Quantification was carried out by a Qubit 2.0 Fluorometer (Thermo Fisher Scientific, Waltham, MA, USA) and the dsDNA BR Assay Kit (Thermo Fisher Scientific, Waltham, MA, USA). The Nextera XT DNA Library Preparation Kit (Illumina, San Diego, CA, USA) was used for library preparation, and paired-end sequencing with a read length of 2 × 300 base pairs was conducted on a NextSeq 2000 device according to the manufacturer’s instructions (Illumina, San Diego, CA, USA). Raw reads were trimmed with Trimmomatic v0.36 on default parameters. SPAdes (version 3.11) and SeqSphere  +  (version 5.1.0) were used for the read assembly. Contigs with a coverage of less than 5× and a length of less than 200 bp were filtered out [62].

Isolates sequenced by the Berlin group were lysed using lysostaphin, proteinase K, and RNAse, followed by DNA purification using the QIAamp^®^ DNA Mini Kit (QIAGEN, Hilden, Germany). The libraries for WGS were prepared using the Nextera XT library preparation kit (Illumina Inc., San Diego, CA, USA) according to the manufacturer’s instructions. The 2 × 300 bp paired-end sequencing in 30-fold multiplexes was performed on the Illumina MiSeq platform. The genome sequences were de novo assembled using Newbler (Roche, Basel, Switzerland) and SPAdes v3.12.0 [66].

### 5.5. Oxford Nanopore Sequencing

Representative isolates of ST1 and ST1660, as well as the ST816 and ST8115 isolates (see above and Supplemental File S2a), were subjected to whole genome sequencing by Nanopore technology as described previously [62].

Briefly, for genome sequencing of all strains, one MinION flow cell (FLO-MIN114, R10.4.1, Oxford Nanopore Technologies (ONT), Oxford, UK) was used. Library preparation was performed using the Native Barcoding Kit 24 V14 (SQK-NBD114.24; Oxford Nanopore Technologies). DNA size selection and clean-up were performed using Agencourt AMPure XP beads (Beckman Coulter GmbH, Krefeld, Germany) at a 1:1 (*v*:*v*) ratio prior to library preparation. DNA nicks and ends were repaired using the NEBNext FFPE DNA Repair Mix and NEBNext Ultra II End Repair/dA-Tailing Module (New England Biolabs, Ipswich, MA, USA) with triple the recommended incubation time. Barcodes were ligated to the dA-prepared DNA ends before sequencing adapter ligation, followed by additional AMPure bead clean-ups. At the start of sequencing, flow cells were quality-checked for a minimum of 1200 active pores, and 40–60 ng of genomic DNA per strain (measured using Qubit 4 Fluorometer; ThermoFisher Scientific, Waltham, MA, USA) was loaded. Sequencing was run for 72 h using MinKNOW software (v22.05.5). The Dorado basecaller (version 0.7.2; Oxford Nanopore Technologies) was used to translate MinION raw reads (POD5) into quality-tagged sequence reads, applying the barcode trimming option with model dna_r10.4.1_e8.2_400bps_sup. Flye software (v2.8.3) was used to assemble each strain’s quality-tagged sequence reads into complete, circular contigs. Assemblies were polished in two stages: first, through four iterative rounds of Racon (v1.4.21) with parameters (-m 8 -x 6 -g 8 -w 500) and then using Medaka (v1.7.1) with models r1041_e82_400bps_sup_v4.3.0.

For the ST1 isolate IMT39173, sequencing initially yielded only a fragmented genome consisting of several contigs, which could be attributed to the presence of five prophages. To obtain longer reads, the high molecular weight DNA kit (Macherey-Nagel, Düren, Germany) was used for DNA extraction according to the manufacturer’s instructions. As recommended, enzymatic lysis with lysozyme was performed at the beginning of this protocol. This strain was subsequently repeated on another flow cell using the same protocol as described above.

### 5.6. Spa and MLST Typing

*Spa* and MLST sequence types were deduced from whole genome sequences using freely accessible websites https://spatyper.fortinbras.us/ for *spa* typing and https://pubmlst.org/bigsdb?db=pubmlst_saureus_seqdef&page=sequenceQuery for MLST (both accessed on 27 November 2024).

### 5.7. Phage Induction and Phage DNA Preparation

Phage induction was performed as previously described [25,67,68]. Briefly, bacterial cultures were inoculated overnight in sterile 2 × TY medium and cultured at 37 °C until the middle of the exponential growth phase (Table 3). Mitomycin C (Roche, Basel, Switzerland) was added at a final concentration of 0.5 μg/mL, and cultivation continued at 30 °C until the optical density (OD at 600 nm) began to decrease compared with the previous measurement point (Table 3). The lysates were centrifuged at 4 °C and 3000× *g*, and the supernatant was neutralised with 0.1 N NaOH and filtered using a 0.20 µm cellulose acetate (CA) membrane filter (Sartorius, Göttingen, Germany). To isolate phage DNA (p-DNA), the supernatant was first treated with 5 µg/mL DNAse I (New England Biolabs GmbH (NEB), Frankfurt a.M., Germany) and 5 µg/mL RNAse (Sigma-Aldrich, St. Louis, MO, USA) for 1 h at 37 °C and 300 rpm. Then, 20 mM EDTA, 50 µg/mL proteinase K, and 0.5% SDS were added sequentially and incubated for another hour at 65 °C and 300 rpm. Phenol–chloroform extraction was then performed as previously described [69]. Phase-lock gel light tubes (Quantabio, Beverly, NJ, USA) were used in each step for better separation of the phases. Finally, the isolated DNA was concentrated in a SpeedVac vacuum concentrator (Eppendorf, Hamburg, Germany) at 1400 rpm and room temperature (20 °C) for 25 min. The final concentration was measured using the Qubit 4 fluorometer (ThermoFisher Scientific, Waltham, MA, USA) according to the manufacturer’s instructions.

### 5.8. Sequencing of Phage Preparations

Whole genome sequencing of six phage preparations was conducted using the Oxford Nanopore Technologies MinION platform. Library preparation for the preparations from strains IMT39173, IMT37083, V641, and 17CS1042 followed the manufacturer’s ligation sequencing gDNA protocol with the Native Barcoding Kit 24 V14 (SQK-NBD114.24; Oxford Nanopore Technologies). Prior to library preparation, an Agencourt AMPure XP purification step was performed at a 1:1 (*v*/*v*) ratio. DNA repair and A-overhang generation were carried out in a single step using the NEBNext FFPE DNA Repair Mix and the NEBNext Ultra II End Repair/dA-Tailing Module (New England Biolabs, Ipswich, MA, USA), with extended incubation times to improve performance. A second purification step was implemented, enabling a barcoding step prior to adapter ligation. Libraries were quantified using a Qubit 4 Fluorometer (ThermoFisher Scientific) and loaded onto the flow cell (FLO-MIN114; R10.4.1; Oxford Nanopore Technologies).

Due to the low concentration of gDNA from the phage preparation of strain V353, the Rapid Sequencing DNA V14-barcoding Kit (SQK-RBK114.24; Oxford Nanopore Technologies) was used for library preparation. Similar to the other samples, only one AMPure XP purification step was included prior to library preparation. This protocol omits the initial DNA repair and A-overhang generation steps, as the rapid barcodes are added via transposase activity. Following a subsequent AMPure XP purification, adapters were ligated to the DNA, and the libraries were loaded onto the flow cell.

For both phage sequencing runs, the Dorado basecaller (version 0.7.2; Oxford Nanopore Technologies) was used to translate MinION raw reads (POD5) into quality-tagged sequence reads, applying the barcode trimming option with model dna_r10.4.1_e8.2_400bps_sup. Flye software (v2.8.3) was used to assemble each phage’s quality-tagged sequence reads into a complete, circular chromosome. Assemblies were polished in two stages: first, through four iterative rounds of Racon (v1.4.21) with parameters -m 8 -x 6 -g 8 -w 500 and then using Medaka (v1.7.1) with model r1041_e82_400bps_sup_v4.3.0.

### 5.9. Transmission Electron Microscopy

Negative staining was carried out on the phage preparations as previously described [25,67,68]. Briefly, copper grids filmed with formvar, coated with carbon, and hydrophilized by glow discharge were placed on 30 µL drops of phage preparations for 30 min. After washing with distilled water, they were placed on a drop of 1% uranyl acetate for 1 min for contrasting. Grids were examined in a transmission electron microscope (Tecnai 12, FEI Deutschland GmbH, Dreieich, Germany), and micrographs of approximately 30 phage particles were taken with a digital camera (TEMCAM FX416, TVIPS, Gauting, Germany). Particle size was measured using the EM-Measure software (version from 2021-05-19, TVIPS).

## Figures and Tables

**Table 1:** Gene content of the *lukP/Q* (pro-) phages of study strains and reference sequences. This is an abridged version, with genes for “hypothetical or putative proteins” being omitted unless they were used as markers for assignment to groups. For a complete version including exact gene positions, see Supplemental File S4.

Gene ID	Description	Allele as in Locus Tag	JRA307 (CC1)	IMT39173 (CC1)	NCTC5663 (CC350)	Phage from 3711	IMT37083 (CC350)	59-071 (CC398)	V353 (CC816)	V0641 (CC8115)	Marker for Group…
			Group 1	Group 1	Group 2	Group 2	Group 2	Group 2	Group 3	Group 3	
*int1* (*lip2=geh*)	Site-specific integrase, lysogeny module	SACOL0318	X	X	X	X	X	X	X	X	1, 2, 3
Q6GAQ4	putative phage protein, Na-K-ATPase	MW1441							X	X	3
-	hypothetical phage protein	MW1440							X	X	3
Q99T51	DUF2871 domain-containing protein	A7971_09875							X	X	3
*pemK*	type II toxin-antitoxin system	SAJRA307_03420	X	X							1
-	hypothetical phage protein	SAJRA307_03430	X	X							1
A6U3A7	lysogeny-assoc. hypoth. protein	SACOL0320			X	X	X	X			2
*xrep*	XRE family regulatory protein/helix-turn-helix transcript. repressor	MW1936	X	X							
		phi29-ORF022				X		X			
		SAB1757							X	X	3
		SAOV_1074c/1970			X		X				
*treG*	helix-turn-helix transcriptional regulator	LB315_12085 or SAOV_1075			X	X		X			
		MW1934	X	X							
		NCTC5663_00871							X	X	
-	putative DNA-binding protein	SAB1756c							X	X	3
-	hypothetical phage protein	MW1933, SAS1916	X	X							1
*ant*	antirepressor	SAB1755, NCTC6131_00928				X		X	X	X	
		MW1932, SAS1914	X	X							
*ant2*	hypothetical phage antirepressor	KMD47_gp08, SAJRA307_03490	X	X	X		X				
-	hypothetical phage protein	MW1929, SAS1912	X	X							1
*dbp*	hypothetical DNA-binding protein	SACOL0333, SAOV_0285	X	X	X	X	X	X	X	X	
*treG*	hypothet. transcriptional regulator	CA347_1950						X			
*ssbP1*	single-stranded DNA-binding protein, replication module	SAJRA307_03590	X					X			
		SIO73104				X					
*ssbP2*	single-stranded DNA-binding protein, replication module	SAJRA307_03600	X								
		SIO73103 or C9J86_00295				X		X			
*ssbP*	single-stranded DNA-binding protein, replication module	RU53_596			X				X	X	
		SPphiMR11_gp14		X			X				
*dnaD2a*	putative replisome-organizer, replication module	SAJRA307_03620	X								
		SaO11_01748						X			
*dnaD2b*	DnaD domain protein	SAB1743c			X	X	X		X	X	
*dnaC1=istB1*	ATP/GTP binding DNA-replication-protein	SaO11_01747, RU53_600		X				X			
*dnaC2*	ATP/GTP binding protein, replication module	phi-55-ORF016	X								
*dbp*	phage protein/helicase loader	SACOL0342			X	X	X		X	X	
*dhlC-2a*	helicase 2, replication module	SACOL0343			X	X	X		X	X	
*sri*	replication inhibitor, replication module	SAJRA307_03640	X	X				X			
*rusA*	resolvase/endodeoxyribonuclease	SAOV_0297	X	X							1
		SAJRA307_03670	X	X	X			X			
*xrep*	XRE family regulator/repressor	SACOL0349							X	X	3
*dbp*	putative polymerase/phi PVL ORF50-like protein, replication module	AOZ05_RS04675							X	X	3
		SAAV_2043				X	X				
		SACOL0350						X			
		SAJRA307_03680	X	X	X						
*gnaT*	hypothetical phage protein/GNAT family acetyltransferase	SAJRA307_03720	X	X							
		SAOV_0304 or SARLGA251_08010			X	X	X				
*dut*	dUTP pyrophosphatase, replication module	SAJRA307_03750	X	X				X			
		MW1415 or SAOV_1092			X	X	X				
		SAS0919							X	X	3
-	hypothetical phage protein	MW1414							X	X	3
-	hypothetical phage protein	SACOL0360	X	X							1
*rinB*	transcript. activator RinB, replication module	MW1916, SAJRA307_03780	X	X	X	X	(X)	X	X	X	
-	hypothetical phage protein	UG86_10335			X	X	X	X			2
-	hypothetical phage protein	MW1912, SAS1895			X	X	X	X			2
-	nucleoside triphosphate pyrophosphohydrolase	MW1911			X	X	X	X			2
-	hypothetical phage protein	SAJRA307_03800	X	X							1
-	hypothetical phage protein	SAJRA307_03810	X	X							1
*rinA*	transcript. activator, replication module	SA1780							X	X	3
*ycfA*	HicA family protein/addiction module toxin	SAJRA307_03820	X	X							1
-	HicB family protein	SAJRA307_03830	X	X							1
*nuc*	HNH endonuclease family protein	SAJRA307_03840	X	X							1
		MW1910			X	X	X	X			2
		SA1779							X	X	3
*terS*	terminase small subunit, packaging module	MW1909			X		X				
		SAOV_1932c				X		X			
-	hypothet. protein terminase small subunit?	SA1778, SAXN108_0348	X	X					X	X	
*terL*	terminase large subunit, packaging module	SA1777	X	X					X	X	
		MW1908			X	X	X	X			2
*port*	portal protein, packaging module	SAOV_1102	X	X							1
		MW1906			X	X	X	X			2
		SAMSHR1132_18060							X	X	3
-	phi PVL ORF3-like hypothet. membrane protein	MW1907			X	X	X	X			2
*clpP*	prohead/head maturation protease	MW1905, SAS1888			X	X	X	X			2
		SAJRA307_03880	X	X							1
		SAMSHR1132_18050							X	X	3
*macp*	major capsid-like protein	A0EWZ3	X	X							1
		MW1904			X	X	X	X			2
		V353-specific							X	X	3
-	phi PVL orf 8-like head protein	MW1903			X	X	X	X			2
-	hypothetical phage protein	SAMSHR1132_18030							X	X	3
-	hypothetical phage protein	SAJRA307_03900	X	X							1
*htcp*	head-tail connector/DNA packaging protein	SAJRA307_03910	X	X							1
		MW1902			X	X	X	X			2
		SAMSHR1132_18020							X	X	3
*hdtA*	head-tail adaptor protein, head module	MW1901			X	X	X	X			2
		SAJRA307_03920	X	X							1
		SAMSHR1132_18010							X	X	3
-	HL97 gp10 protein, head module	MW1900			X	X		X			2
		SAXN108_0356	X	X					X	X	
*matp*	major tail protein, tail module	SAJRA307_03950	X	X							1
		MW1898			X	X	X	X			2
		SA1768							X	X	3
-	hypothetical phage protein	SAJRA307_03960	X	X							1
-	hypothetical phage protein	MW1897, NCTC5663_00336			X	X	X	X			2
-	hypothetical phage protein	MW1896			X	X	X	X			2
*tmpM*	tail tape measure protein, tail module	SAJRA307_03990	X	X					X	X	
		MW1895, SAS1878			X	X	X	X			
*pep*	phage tail peptidase/minor structural protein	MW1893, SAOV_1117	X	X	X	X	X	X	X	X	
*sea*_320E_	phage associated enterotoxin	SAOV_1120							X	X	3
*holA*	holin	SAB0780	X	X	X	X	X	X	X	X	
*amidase*	endolysin/amidase, lysis module	MW1886	X	X	X	X	X	X	X	X	
*lukP*	equine/caprine leukocidin S component	SAJRA307_04070	X	X	X	X	X	X	X	X	1, 2, 3
*lukQ*	equine leukocidin F component	SAJRA307_04080	X	X	X	X	X	X	X	X	1, 2, 3
*scn-eq*	equine staphylococcal complement inhibitor	SAJRA307_04090	X	X	X	X	X	X	X	X	1, 2, 3
*hxlB*	lysis-associated hypoth. protein	SPV-80A_gp72	X	X	X	X	X	X	X	X	1, 2, 3

**Table 2:** Morphological characteristics of induced phages (MV, mean value; STD, standard deviation). V641 is not shown as phage particles were not complete, see text.

Strain ID	General Shape (Number of Particles)	Head Diameter in nm		Head Length in nm		Tail Diameter in nm		Tail Length in nm		Baseplate in nm
		MV ± STD	Range	MV ± STD	Range	MV ± STD	Range	MV ± STD	Range	Range
IMT39173	Icosahedral (*n* = 15)	56 ± 4	49–61	55 ± 4	48–63	11 ± 2	8–14	222 ± 19	188–258	14 × 24
	Prolate-thick (*n* = 11)	56 ± 3	53–62	91 ± 4	82–97	10 ± 1	9–11	284 ± 16	267–310	20 × 36
	Prolate-thin (*n* = 2)	--	40, 41	--	88, 91	--	10, 10		258, 280	21 × 25
IMT37083	Icosahedral (*n* = 28)	51 ± 3	47–58	51 ± 2	47–54	11 ± 1	8–15	223 ± 21	197–259	16 × 29
	Elongated (*n* = 12)	51 ± 2	48–53	57 ± 3	53–61	10 ± 2	8–15	234 ± 73	166–422	17 × 20
V353	Prolate-thick (*n* = 20)	56 ± 5	50–62	94 ± 7	87–111	10 ± 1	9–12	297 ± 30	231–318	18 × 31
	Prolate-thin (*n* = 15)	41 ± 3	37–47	91 ± 3	84–94	11 ± 1	8–13	265 ± 53	143–296	21 × 33
17CS1042	Icosahedral-large (*n* = 20)	54 ± 3	50–57	55 ± 4	50–64	11 ± 1	9–12	215 ± 70	126–390	18 × 25
	Icosahedral-small (*n* = 9)	48 ± 2	45–51	48 ± 4	42–53	10 ± 1	9–11	205 ± 29	175–233	17 × 20

**Table 3:** Overview of cultivation parameters of the strains analysed: OD and cultivation time until induction (columns 2–3), OD and time when cultivation was discontinued (columns 4–5), as well as the concentration of the isolated phage DNA (column 6).

Strain ID	Induction		Cultivation Finished		Final Concentration
	OD	Time [min]	OD	Time [min]	c [ng/µL]
IMT37083	1.19	150	1.96	330	40.80
IMT39173	0.55	150	0.55	330	21.10
V353	0.45	60	3.75	240	28.40
V641	0.68	150	1.29	330	7.95
17CS1042	0.56	150	0.12	330	11.55

## Data Availability

All relevant data are provided as Supplementary Materials. The Nanopore sequences of the genomes can be accessed at GenBank (accession numbers, CP176564-CP176569). The Illumina sequences of the Austrian strains are submitted, BioProject PRJNA1194107. The accession numbers of the genome sequences of the other strains are listed in Supplemental File S2a.

## Supplementary Materials

The following supporting information can be downloaded at https://www.mdpi.com/article/10.3390/toxins17010020/s1. Supplemental File S1: Phage integration sites and localisation of leukocidin genes in the genome of *S. aureus*. Supplemental File S2: Genome sequences of study strains and previously published sequences of equine strains of *S. aureus*. S2a, general characteristics; S2b, *lukP/Q* phages; S2c, phages carrying the equine kinase gene; S2d, resistance genes. Supplemental File S3: Sequences of phages/prophages discussed herein. All are displayed with their respective integrase genes first. Sequences marked with an asterisk (*) are those that were used for the construction of the Tables and the other Supplementary files. Supplemental File S4: Gene content of the *lukP/Q* phages/prophages of studied isolates and reference sequences. Supplemental File S5: Gene content of the *sak*_phi-42e_ phages/prophages of studied isolates and reference sequences.
