# Supplementary material for: Characterisation of Staphylococcus aureus Strains and Their Prophages That Carry Horse-Specific Leukocidin Genes lukP/Q
Source: Toxins (Basel). 2025 Jan 3;17(1):20. doi: 10.3390/toxins17010020 (PMC11769447; doi:10.3390/toxins17010020)

Phage integration sites and localisation of leukocidin genes in the genome of *S. aureus*.

Horse strains are described in this paper. The CC522 goat isolate is CP138360.1. CC49 and CC1956 beaver strains are CP084107 and CP084892, respectively; CC705 cattle strains are AJ938182, LS483300, LR134088; Human PVL strains are, for instance, BA000033, CP000730, CP003194, CP002114, CP010526; ovine CC133 include CP001996, LR134305, LR134271, LR134090; cow strains of CC479 include CP155061 and CP155058; and the squirrel CC49 strain is CP065355.

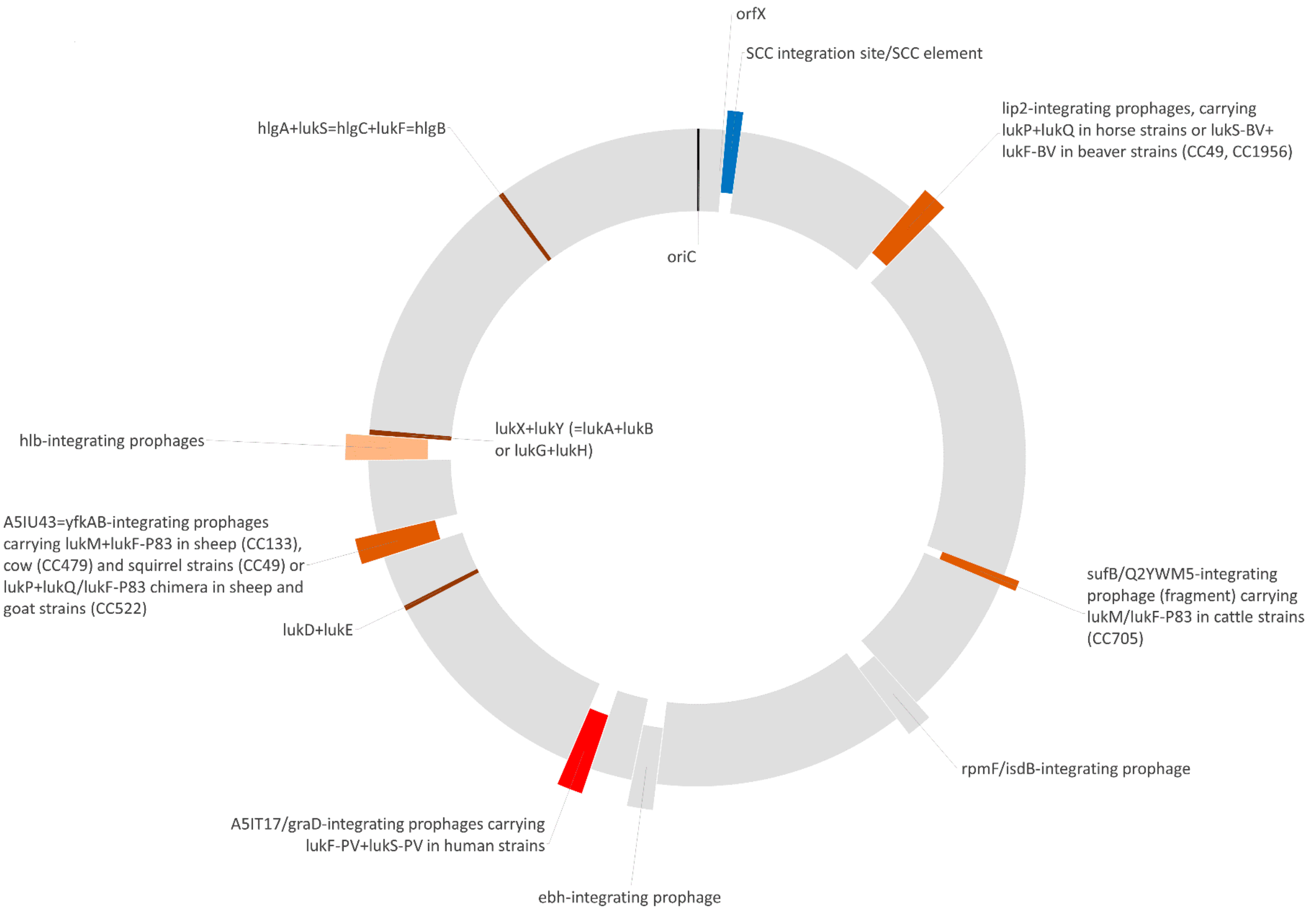

Supplement: Supplementary file 1 [file toxins-17-00020-s001.zip › Supplemental file S1_Leukocidin genes and phage integration sites.pdf]
