# Supplementary material for: Characterisation of Staphylococcus aureus Strains and Their Prophages That Carry Horse-Specific Leukocidin Genes lukP/Q
Source: Toxins (Basel). 2025 Jan 3;17(1):20. doi: 10.3390/toxins17010020 (PMC11769447; doi:10.3390/toxins17010020)
Supplement: Supplementary file 1 [file toxins-17-00020-s001.zip › Supplemental file S4_Gene Content of lukPQ Phages.pdf]

Gene content of the *lukP/Q* phages/prophages of studied isolates and reference sequences.

Gene ID	Description	Locus tags	AP019751.1 JRA307 (CC1)	IMT39173 (CC1)	LS483317.1 NCTC5663 (CC350)	LT671578.1 prophage from strain 3711	IMT37083 (CC350, ST1660)	Strain 59-071 AAFLK 010000001.1 (CC398)	V353 (CC816)	V0641 (CC8115)	Used as marker for Group....
			Group 1	Group 1	Group 2	Group 2	Group 2	Group 2	Group 3	Group 3	
***Genus classification according to "What The Phage"* (https://github.com/replikation/What_the_Phage):**			*Biseptima*	*Biseptima*	N/A	N/A	N/A	N/A	N/A	N/A	
**phi-int1 (lip2=geh)**	site-specific integrase, lysogeny module	SACOL0318	1..1206	1..1206	1..1206	1..1206	1..1206	277083..278288	1..1206	1..1206	**1,2,3**
**phi-PVL-ORF30**	putative Na-K-ATPase/phi-PVL-ORF30-like protein/hypothetical protein, lysogeny module	NCTC5663_00278			1311..1928		1311..1928				
		KMD47_gp02				1316..1930		278398..279012			
**phi-Q6GAQ4**		MW1441							1316..1939	1316..1939	**3**
**-**	hypothetical phage protein	MW1440							1926..2030	1926..2030	**3**
**phi-Q99T51**	putative lipoprotein/DUF2871 domain-containing protein	A7971_09875							2424..3038	2424..3038	**3**
**phi-pemK**	type II toxin-antitoxin system PemK/MazF family toxin	SAJRA307_03420	1382..1921	1382..1921							**1**
**-**	hypothetical phage protein	SAJRA307_03430	2121..2609	2121..2609							**1**
**-**	hypothetical phage protein	phi-3A-ORF130				1927..2052		279009..279135			
**-**	hypothetical phage protein	NCTC5663_00279			1935..2087		1935..2087				
**phi-A6U3A7**	lysogeny-assoc. hypothetical phage protein	SACOL0320/SPV-80A_gp05n			2144..2326	2109..2291	2144..2326	279192..279374			**2**
**-**	hypothetical phage protein	NCTC5663_00281			2594..2713		2465..2582				
**-**	putative phage protein	SAEMRSA15_19220			2700..2855		2569..2724				
**-**	HIRAN domain protein/hypothetical phage protein	SAOV_1971/SAXN108_0311					2798..3652				
**-**	hypothetical phage protein, related to DUF4355 scaffold protein	SIO73112				2424..3044		279507..280127			
**phi-xrep**	XRE family regulatory protein/helix-turn-helix transcriptional repressor	MW1936	2666..3436	2666..3436							
		phi-29-ORF022				3095..3727		280178..280810			
		SAB1757							3095..3736	3095..3736	**3**
		SAOV_1074c			2867..3574						
		SAOV_1970					3664..4347				
**phi-DUF0739**	DUF739 family protein/phage DNA-binding protein	MW1935	3595..3819	3595..3819							
		SAOV_1969c					4544..4786				
**phi-treG**	helix-turn-helix transcriptional regulator	LB315_12085				3881..4108		280964..281191			
		MW1934	3837..4097	3837..4097							
		NCTC5663_00871							7252..7482	7252..7482	
		SAOV_1075			3745..3984						

Gene ID	Description	Locus tags	AP019751.1 JRA307 (CC1)	IMT39173 (CC1)	LS483317.1 NCTC5663 (CC350)	LT671578.1 prophage from strain 3711	IMT37083 (CC350, ST1660)	Strain 59-071 AAFLK 010000001.1 (CC398)	V353 (CC816)	V0641 (CC8115)	Used as marker for Group....
			Group 1	Group 1	Group 2	Group 2	Group 2	Group 2	Group 3	Group 3	
-	putative phage-related protein/DNA-binding protein	SAB1756c							3919..4134	3919..4134	**3**
-	hypothetical phage protein	MW1933/SAS1916	4121..4660	4121..4660							**1**
**phi-ant**	antirepressor	SAB1755/NCTC6131_00928				4131..4901		281214..281984	4150..4938	4150..4938	
		MW1932/SAS1914	4717..5466	4717..5466							
**phi-ant2**	hypothetical phage protein	KMD47_gp08/SAJRA307_03490	5479..5922	5479..5922	3997..4440		4799..5242				
**phi-Q931J5**	phi PVL orf 35-like protein, lysogeny module	SAA6159_01927				4918..5112		282001..282195	4955..5149	4955..5149	
**phi-DUF2513**	DUF2513 domain-containing protein	MS7_2025				5107..5463		282190..282546	5144..5500	5144..5500	
-	conserved hypothetical phage protein	MW1930/SAAV_2057/KMD47_gp09	5937..6077	5937..6077	4455..4595		5257..5397				
-	hypothetical phage protein	SAOV_1966/SAXN108_0316			4588..4797		5390..5599				
-	hypothetical phage protein	SIO73108				5441..5704		282524..282787	5478..5741	5478..5741	
		SIO73108-trnc/SACOL0331			4854..5045		5656..5847				
-	hypothetical phage protein	SACOL0332/NCTC5663_00289			5047..5274	5706..5933	5849..6076	282765..283016	5743..5970	5743..5970	
-	hypothetical phage protein	MW1929/SAS1912	6092..6724	6092..6724							**1**
-	hypothetical phage protein	NCTC5663_00290/SAJRA307_03520	6780..7106	6780..7106	5330..5656	5989..6315	6132..6458	283072..283398	6030..6356	6030..6356	
-	ROSA-ORF074/phi PV83 orf 10-like protein	SAJRA307_03530	7139..7321	7139..7321	5689..5871	6348..6530	6491..6673	283431..283613	6389..6571	6389..6571	
-	hypothetical phage protein	SAOV_1963						284011..284223	6969..7181	6969..7181	
**phi-dbp**	DNA-binding protein /hypothetical phage protein	SACOL0333/SAOV_0285	7351..7614	7351..7614	5901..6164	6560..6823	6703..6966	283643..283906	6601..6864	6601..6864	
**phi-treG**	hypothet. phage protein/hypothet. transcriptional regulator	CA347_1950						284294..284515			
**phi-DUF1270**	DUF1270 family protein, replication or lysogeny module	SACOL0334/UG86_01455			6176..6337		6978..7139				
		KMD47gp15							7466..7627	7466..7627	
		SAS063						284508..284669			
-	hypothet. protein, overlaps phi-DUF1270 but in opposite direction	ROSA-ORF193	7647..7757	7647..7757	6196..6306		6998..7108	284528..284638	7486..7596	7486..7596	
-	hypothetical phage protein	NCTC5663_00294			6430..6759						
-	hypothetical phage protein	SAOV_1961c	7795..8115	7795..8115							
**phi-DUF2482**	putative phage-related protein/DUF2482 family protein	SA1798							7721..8023	7721..8023	
**phi-DUF1108**	lysogeny-assoc. DUF1108 family protein	MW1926/SAJRA307_03560/ NCTC5663_00874	8209..8469	8209..8469	6740..7000	7090..7350	7230..7490	284771..285031	8028..8288	8028..8288	
**phi-DUF2483**	lysogeny-assoc. DUF2483 family protein	SACOL0337			7010..7231				8298..8519	8298..8519	
-	ATP-binding protein, replication module	phi-52A-ORF016			7224..8003				8512..9291	8512..9291	

Gene ID	Description	Locus tags	AP019751.1 JRA307 (CC1)	IMT39173 (CC1)	LS483317.1 NCTC5663 (CC350)	LT671578.1 prophage from strain 3711	IMT37083 (CC350, ST1660)	Strain 59-071 AAFLK 010000001.1 (CC398)	V353 (CC816)	V0641 (CC8115)	Used as marker for Group....
			Group 1	Group 1	Group 2	Group 2	Group 2	Group 2	Group 3	Group 3	
-	hypothetical phage protein SAAV_2053	SAAV_2053	8477..8713	8477..8713				285039..285275			
-	hypothetical phage protein, Gp157 family protein	SAOV_1958c	8706..9185	8706..9185		7364..7843	7505..7984	285268..285747			
-	AAA-ATPase	phi-55-ORF017		9185..9958			7984..8757				
**phi-ssbP1**	single-stranded DNA-binding protein, replication module	SAJRA307_03590	9224..9823					285786..286385			
		SIO73104				8067..8483					
**phi-ssbP2**	single-stranded DNA-binding protein, replication module	SAJRA307_03600	9823..10248								
		SIO73103				8483..8908					
		C9J86_00295						286385..286813			
**phi-ssbP**	single-stranded DNA-binding protein, replication module	RU53_596			8034..8585				9321..9875	9321..9875	
		SPphiMR11_gp14		9988..10506			8787..9305				
-	hypothetical phage protein	phi-55-ORF167			8097..8216						
-	hypothetical phage protein	phi-69-ORF076			8380..8577						
**phi-DUF0968**	putative HNHc nuclease/DUF968 domain-containing protein	NCTC5663_00299			8598..9269				9888..10559	9888..10559	
		SACOL0340/SAJRA307_03610	10262..10957					286827..287523			
		SIO73102				8922..9596					
		SPphiMR11_gp15					9317..9991				
**phi-dnaD2a**	putative replisome-organizer, replication module	SAJRA307_03620	10929..11750								
**=Q4ZAY9**		SaO11_01748						287531..288298			
**phi-dnaD2b**	DnaD domain protein	SAB1743c			9262..10017	9589..10341	9984..10736		10552..11304	10552..11304	
**phi-DUF4393**	DUF4355 domain-containing phage protein	FIV54_08820		11297..12154							
-	hypothet. phage protein	phi-53-ORF162		11188..11304							
**phi-Q4ZAK4**	helix-turn-helix family protein	NW973_06205		12218..12931							
**phi-dnaC1=istB1**	ATP/GTP binding protein, phage DNA-replication-protein DnaC	SaO11_01747,RU53_600		12941..13720				288308..289081			
**phi-dnaC2**	ATP/GTP binding protein, replication module	phi-dnaC2_phi-55-ORF016	11763..12548								
**phi-dbp**	phage protein/helicase loader	SACOL0342			10017..10373	10341..10697	10736..11092		11304..11660	11304..11660	
**phi-dhlC-2a**	helicase 2/ 1242 nt, replication module	SACOL0343			10370..11611	10694..11935	11089..12330		11657..12898	11657..12898	
**phi-sri**	staphylococcal replication inhibitor, replication module	SAJRA307_03640	12545..12703	13714..13872				289075..289233			
-	putative phage related protein	SACOL0344			11608..11823	11932..12147	12327..12542		12895..13110	12895..13110	

Gene ID	Description	Locus tags	AP019751.1 JRA307 (CC1)	IMT39173 (CC1)	LS483317.1 NCTC5663 (CC350)	LT671578.1 prophage from strain 3711	IMT37083 (CC350, ST1660)	Strain 59-071 AAFLK 010000001.1 (CC398)	V353 (CC816)	V0641 (CC8115)	Used as marker for Group….
			Group 1	Group 1	Group 2	Group 2	Group 2	Group 2	Group 3	Group 3	
**phi-DUF3269**	DUF3269 family protein, replication module	SACOL0345	12716..12937		11827..12048	12150..12371	12546..12767		13113..13334		
**phi-DUF1064**	DUF1064 domain-containing protein, replication module	SACOL0347				12381..12785	12777..13181		13345..13749	13346..13750	
		SAOV_1085			12059..12463			289479..289883			
**phi-rusA**	bacteriophage resolvase endodeoxyribonuclease	SAOV_0297	12947..13354	14115..14522							1
**phi-DUF3113**	DUF3113 family protein, replication module	SACOL0348				12790..12975	13186..13371		13754..13939	13755..13940	
		SAJRA307_03670	13354..13539	14522..14707	12468..12653			289888..290073			
**phi-xrep**	XRE family regulator/helix-turn-helix transcriptional repressor	SACOL0349							13940..14197	13941..14198	3
**phi-dbp**	putative polymerase/phi PVL orf 50-like protein, replication module	AOZ05_RS04675							14209..14571	14210..14572	3
		SAAV_2043				13246..13605	13372..13731				
		SACOL0350						290074..290430			
		SAJRA307_03680	13540..14157	14708..15325	12654..13270						
**phi-DUF3310**	DUF3310 domain-containing protein/hypothet. nucleotide kinase	SAS0912, MW1422/KMD47_gi13	14157..14411	15325..15579	13270..13524				14568..14822	14569..14823	
**phi-DUF1270**	DUF1270 family protein, replication module	MW1420/NCTC5663_00310	14417..14659	15585..15827	13530..13772			290434..290676		14829..15071	
		SAOV_0301				13606..13854	13732..13980				
	hypothetical phage protein, replication module	SACOL0353/SAJRA307_03710	14673..15080	15841..16248	13786..14193	13868..14278	13994..14401				
**phi-gnaT**	hypothetical phage protein/GNAT family acetyltransferase	SAJRA307_03720	15080..15436	16248..16604							
		SAOV_0304			14193..14468	14278..14553	14401..14676				
		SARLGA251_08010						291528..291812			
**phi-DUF1024**	DUF1024 family protein, replication module	SAJRA307_03730	15429..15665	16597..16833	14461..14712	14546..14797	14669..14917	291805..292053	15085..15330	15086..15331	
-	hypothetical phage protein	SAJRA307_03740	15655..15780	16823..16948							
-	hypothetical phage protein	MW1418			14702..14875						
-	hypothetical phage protein	MW1417			14876..15157						
-	hypothetical phage protein	MW1416			15158..15319	14961..15122					
**phi-dut**	dUTP pyrophosphatase, replication module	SACOL0357/SAJRA307_03750	15889..16422	17057..17590				292046..292555			
		MW1415			15334..15867	15137..15673					
		SAS0919							15327..15863	15328..15864	3
		phi-dutSAOV_1092					14910..15443				
-	hypothetical phage protein	NCTC5663_00894				15710..15955					
-	hypothetical phage protein	MW1414							15909..16145	15910..16146	3

Gene ID	Description	Locus tags	AP019751.1 JRA307 (CC1)	IMT39173 (CC1)	LS483317.1 NCTC5663 (CC350)	LT671578.1 prophage from strain 3711	IMT37083 (CC350, ST1660)	Strain 59-071 AAFLK 010000001.1 (CC398)	V353 (CC816)	V0641 (CC8115)	Used as marker for Group....
			Group 1	Group 1	Group 2	Group 2	Group 2	Group 2	Group 3	Group 3	
-	hypothetical phage protein	phi-71-ORF027					15625..16083				
**phi-DUF1381**	DUF1381 domain-containing protein, replication module	SACOL0358	16459..16665	17627..17833	15904..16110	15952..16158		292622..292828	16142..16348	16143..16349	
-	hypothetical phage protein	SACOL0360	16662..16865	17830..18033							**1**
**phi-rinB**	transcriptional activator RinB, replication module	MW1916/SAJRA307_03780<br>MW1916/SAJRA307_03780-trnc	16858..17007	18026..18175	16107..16256	16155..16304	 16135..16245	292825..292974	16345..16494	16346..16495	
**Q4ZCN3**<br>**=DUF1514**	DUF1514 family protein/conserved putative phage protein	SAJRA307_03790<br>MW1913	17007..17207	18175..18375	 16268..16456	 16316..16504	 16257..16445	 292986..293174	 16506..16694	 16507..16695	
-	hypothetical protein, UG86_10335	UG86_10335			16360..16572	16408..16620	16349..16561	293078..293290			**2**
-	hypothetical phage protein	MW1912/SAS1895			16479..16940	16527..16988	16468..16929	293197..293667			**2**
-	putative nucleoside triphosphate pyrophosphohydrolase	MW1911			17055..17507	17103..17555	17044..17496	293782..294234			**2**
-	hypothetical phage protein	SAJRA307_03800	17230..17658	18398..18826							**1**
-	hypothetical phage protein	SAJRA307_03810	17618..17725	18786..18893							**1**
**phi-rinA**	RinA family transcriptional activator, replication module	SA1780							16722..17138	16723..17139	**3**
**phi-ycfA**	YcfA-like protein/HicA family protein/addiction module toxin	SAJRA307_03820	17829..18014	18997..19182							**1**
-	HicB family protein	SAJRA307_03830	18049..18444	19217..19612							**1**
**phi-nuc**	HNH endonuclease family protein	SAJRA307_03840<br>MW1910/UG86<br>SA1779	18495..18869	19663..20037	 17523..17867	 17571..17915	 17512..17856	 294250..294594	 <br>17373..17669	 <br>17374..17670	**1**<br>**2**<br>**3**
-	putative phage protein	SAR2065a	18790..18903	19958..20071					17663..17776	17664..17777	
**phi-terS**	terminase small subunit, packaging module	MW1909<br>SAOV_1932c			17996..18463	 18044..18514	17985..18452	 294723..295193			
**phi-terS?**	phage protein, possibly a terminase small subunit	SA1778/SAXN108_0348	18926..19270	20094..20438					17799..18143	17800..18144	
**phi-terL**	terminase large subunit, packaging module	SA1777<br>MW1908	19267..20928	20435..22096	 18466..20160	 18514..20208	 18455..20149	 295193..296887	18140..19801	18141..19802	 **2**
**phi-port**	phage portal protein, packaging module	SAOV_1102<br>MW1906<br>SAMSHR1132_18060	20944..22131	22112..23299	 20443..21630	 20491..21678	 20432..21619	 297170..298357	 <br>19816..20964	 <br>19817..20965	**1**<br>**2**<br>**3**
-	phi PVL orf 3-like protein/hypothetical membrane protein	MW1907			20174..20374	20222..20422	20163..20363	296901..297101			**2**

Gene ID	Description	Locus tags	AP019751.1 JRA307 (CC1)	IMT39173 (CC1)	LS483317.1 NCTC5663 (CC350)	LT671578.1 prophage from strain 3711	IMT37083 (CC350, ST1660)	Strain 59-071 AAFLK 010000001.1 (CC398)	V353 (CC816)	V0641 (CC8115)	Used as marker for Group....
			Group 1	Group 1	Group 2	Group 2	Group 2	Group 2	Group 3	Group 3	
**phi-clpP**	phage prohead/head maturation protease	MW1905/SAS1888			21683..22207	21731..22255	21672..22196	298410..298934			**2**
**phi-clpP**	phage prohead/head maturation protease	SA1775/SAJRA307_03880	22115..22852	23283..24020							**1**
**=Q6GF91**		SAMSHR1132_18050							20961..21683	20962..21684	**3**
**phi-macp**	major capsid-like protein, bacteriophage head protein	A0EWZ3	22876..24021	24044..25189							**1**
		MW1904			22295..23542	22343..23590	22284..23531	299022..300269			**2**
		V353-specific							21707..22846	21708..22847	**3**
-	phi PVL orf 8-like protein, head module	MW1903			23578..23736	23626..23784	23567..23725	300305..300463			**2**
-	hypothetical phage protein	SAMSHR1132_18030							22865..23143	22866..23144	**3**
-	hypothetical phage protein	SAJRA307_03900	24044..24328	25212..25496							**1**
**phi-htcp**	head-tail connector protein/DNA packaging protein, head module	SAJRA307_03910	24306..24611	25474..25779							**1**
		MW1902			23745..24077	23793..24125	23734..24066	300472..300804			**2**
		SAMSHR1132_18020							23152..23433	23153..23434	**3**
**phi-hdtA**	head-tail adaptor protein, head module	MW1901			24067..24399	24115..24447	24056..24388	300794..301126			**2**
		SAJRA307_03920	24586..24948	25754..26116							**1**
		SAMSHR1132_18010							23417..23779	23418..23780	**3**
-	HL97 gp10 family protein, head module	MW1900			24399..24776	24447..24824		301126..301503			**2**
		SAXN108_0356	24945..25349	26113..26517					23776..24180	23777..24181	
-	hypothetical phage protein	SAXN108_0357/SAOV_1109	25346..25753	26514..26921					24177..24584	24178..24585	
-	hypothetical phage protein	MW1899			24773..25153	24821..25201	24762..25142	301500..301880			
**phi-matp**	major tail protein, tail module	SAJRA307_03950	25754..26395	26922..27563							**1**
		MW1898			25154..26107	25202..26155	25143..26096	301881..302834			**2**
		SA1768							24585..25226	24586..25226	**3**
-	hypothetical phage protein	SAOV_1111/SAXN108_0359							25352..25492		
-	hypothetical phage protein	SAJRA307_03960	26458..26661	27626..27829							**1**
**phi-Q7A4M4**	putative bacteriophagal protein	SA1767/SAJRA307_03970	26711..27061	27879..28229					25543..25893	25543..25893	
-	hypothetical phage protein	SAJRA307_03980	27088..27249	28256..28417					25920..26081	25920..26081	
-	hypothetical phage protein	MW1897/NCTC5663_00336			26172..26618	26220..26666	26161..26607	302899..303345			**2**
-	hypothetical phage protein	MW1896			26717..26800	26765..26848	26706..26789	303444..303527			**2**
**phi-tmpM1**	tail tape measure protein, tail module	SA300HOU_1959/SAJRA307_03990	27306..31838	28474..33006					26138..30666	26138..30658	

Gene ID	Description	Locus tags	AP019751.1 JRA307 (CC1)	IMT39173 (CC1)	LS483317.1 NCTC5663 (CC350)	LT671578.1 prophage from strain 3711	IMT37083 (CC350, ST1660)	Strain 59-071 AAFLK 010000001.1 (CC398)	V353 (CC816)	V0641 (CC8115)	Used as marker for Group....
			Group 1	Group 1	Group 2	Group 2	Group 2	Group 2	Group 3	Group 3	
**phi-tmpM2**	tail tape measure protein, tail module	MW1895/SAS1878			26877..31508	26925..31556	26866..31512	303604..308232			
**phi-Q8SDK3-tail**	putative bacteriophagal tail protein	SAJRA307_04000/MW1894	31835..33319	33003..34487	31508..32998	31556..33046	31512..33002	308232..309722	30663..32147	30655..32125	
**phi-pep/minor**	phage tail peptidase/phage minor structural protein, tail module	MW1893/SAOV_1117	33443..37120	34611..38288	33122..36799	33170..36847	33126..36803	309846..313523	32271..35948	32249..35925	
	hypothetical phage protein, SAR2047, SAXN108_0365	SAR2047	37110..37262	38278..38430	36789..36941	36837..36989	36793..36945	313513..313665	35938..36090	35915..36067	
**phi-holA**	putative phage protein, possibly holin	SAOV_1914c/LB316_01660	37309..37596	38477..38764	36988..37275		36992..37279	313712..313999	36137..36424	36114..36401	
		SAS1874				37035..37322					
**phi-Q7A4M7**	putative bacteriophagal protein	SAOV_1119							36480..36887	36457..36864	
		MW1890/SA1762				37378..37752					
**Q9MBN6=DUF2951**	DUF2951 domain-containing protein, tail module	SAJRA307_04040	37654..37950	38822..39118	37333..37629		37337..37633	314057..314353			
**entA=sea-320E**	phage associated enterotoxin	SAOV_1120							37342..38121	37319..38101	**3**
**phi-txpA**	putative holin-like toxin/putative phage membrane protein	MW1888	38145..38276	39313..39444	37824..37955	37941..38072	37828..37959		38367..38498	38347..38478	
**-**	hypothetical phage protein	SAA6159_03495	38329..38436	39497..39604	38008..38115		38012..38119	314731..314838			
**phi-holA**	holin	SAB0780	38509..38742	39677..39910	38188..38421	38297..38530	38192..38425	314911..315144	38723..38956	38703..38936	
**phi-amidase**	CHAP domain-containing protein/endolysin/amidase, lysis module	MW1886	38754..39509	39922..40677	38433..39188	38542..39297	38437..39192	315156..315911	38968..39723	38948..39703	
**lukP**	equine/caprine leukocidin S component	NCTC5663_00346/SAJRA307_04070	39948..40883	39922..40677	39627..40562	39735..40670	39631..40566	316349..317284	40161..41096	40141..41076	**1,2,3**
**lukQ**	equine leukocidin F component	NCTC5663_00347/SAJRA307_04080	40885..41865	41116..42051	40564..41544	40672..41652	40568..41548	317286..318266	41098..42078	41078..42058	**1,2,3**
**scn-eq**	equine variant of staphylococcal complement inhibitor, eqSCIN	NCTC5663_00348/SAJRA307_04090	42306..42650	42053..43033	41985..42329	42093..42437	41989..42333	318707..319051	42519..42863	42499..42843	**1,2,3**
**phi-hxlB**	Lysis-associated hypothetical protein	SPV-80A_gp72	42856..43083	43474..43818	42535..42762	42643..42870	42539..42766	319257..319484	43069..43296	43049..43276	**1,2,3**
**-**	hypothetical phage protein	C9J86_06615	42959..43258	44024..44251	42638..42937		42642..42941	319360..319659			
