# Supplementary material for: Characterisation of Staphylococcus aureus Strains and Their Prophages That Carry Horse-Specific Leukocidin Genes lukP/Q
Source: Toxins (Basel). 2025 Jan 3;17(1):20. doi: 10.3390/toxins17010020 (PMC11769447; doi:10.3390/toxins17010020)
Supplement: Supplementary file 1 [file toxins-17-00020-s001.zip › Supplemental file S5_Gene Content of Horse Kinase Prophages.pdf]

Gene content of the *sak*_phi-42e phages/prophages of studied isolates and reference sequences.

Gene ID	Description	Locus tags	A5IT17-integr. prophage from V353 (CC816)	hlb-integr. prophage from JRA307, AP019751 (CC1)	hlb-integr. prophage from IMT39173 (CC1)	Phage 42e, NC_007052.1	hlb-integr. prophage from V641 (CC8115)	sufB-integr. prophage from IMT37083 (CC350)	sufB-integr. prophage from NCTC5663, LS483317 (CC350)
Genus classification according to "What The Phage" (https://github.com/replikation/What_the_Phage):			***Triavirus***	***Triavirus***	***Triavirus***	***Triavirus***	***Triavirus***	***Phietavirus***	***Dubowvirus***
**phi-int (A5IT17)**	integrase of A5IT17-integrating prophages, lysogeny module of Siphoviridae	MW1442	1..1206						
**phi-int (hlb)**	integrase of hlb-integrating prophages, lysogeny module of Siphoviridae	MW1939		1..1038	1..1038	1..1038	1..1038		
**phi-int (sufB)**	integrase of sufB-integrating prophages, lysogeny module of Siphoviridae	SARLGA251_07760						1..1050	1..1050
**phi-DUF3644**	DUF3644 domain-containing putative phage protein	NCTC6131-00923					1216..2184		
**-**	hypothet. protein from Siphoviridae	ST42eORF026		1105..1602	1105..1602	1105..1602			
**-**	hypothet. protein from Siphoviridae	ST42eORF031		1592..2017	1592..2017	1592..2017			
**D2N8G3**	putative protein	SAJRA307_20350		2102..2686	2102..2686	2102..2686			
		NCTC5663_00860							1113..1628
**-**	putative protein	SAB1758							1632..2132
**A6U3A7**	putative bacteriophagal protein, PV83 orf 4-like protein, lysogeny module of Siphoviridae	NCTC5663_00862							2185..2370
**-**	Na-K-ATPase/putative protein, lysogeny module of Siphoviridae	KMD47-gp02	1332..1946						
**-**	hypothet. protein from Siphoviridae	phi-3A-ORF130	1943..2068						
**-**	putative phage protein	MW1439	2179..2574						
**-**	putative lipoprotein/phiSLT ORF144-like protein	MW1438	2603..3037						
**DUF0955**	putative metallo-protease, lysogeny module of Siphoviridae KMD47_gp05/SAAV_2063	KMD47_gp05/SAAV_2063	3055..3516				2216..2680		
**phi-repC**	helix-turn-helix family protein, putative phage regulatory protein, SAS0897	SAS0897					2693..3022		
**phi-xrep**	XRE family regulatory protein/helix-turn-helix transcriptional repressor, lysogeny module of Siphoviridae	NW955_05325					3183..3374		
		MW1436	3529..3852						
		phi-29-ORF022						2917..3549	
		SaO11-00271		2742..3353	2742..3353				
		SARLGA251_07780							2427..3065
		SACOL0321				2742..3461			
**phi-cro/hth**	helix-turn-helix transcriptional Cro regulator, lysogeny module of Siphoviridae	ST42eORF076/ SACOL0322				3603..3821			
**phi-bagd**	putative protein	SACOL0323				3836..4144			
**-**	hypothetical protein, SACOL0324	SACOL0324				4148..4300			
**phi-repC**	putative Cro-like repressor/helix-turn-helix family protein	MW1435/SAAV_2061	4016..4264						
**phi-ant**	anti-repressor, lysogeny module of Siphoviridae	SAR1554	4277..4720						
		AOZ05_RS10860						3953..4705	
		SACOL0325				4301..5092			3917..4708

Gene ID	Description	Locus tags	A5IT17-integr. prophage from V353 (CC816)	hlb-integr. prophage from JRA307, AP019751 (CC1)	hlb-integr. prophage from IMT39173 (CC1)	Phage 42e, NC_007052.1	hlb-integr. prophage from V641 (CC8115)	sufB-integr. prophage from IMT37083 (CC350)	sufB-integr. prophage from NCTC5663, LS483317 (CC350)
**phi-rinB**	transcriptional activator RinB, replication module of Siphoviridae	SAAV_2059	4735..4884						
-	conserved hypothetical phage protein, SAAV_2058	SAAV_2058	4925..5137						
**phi-Q931J5**	putative protein, phi PVL orf 35-like protein, lysogeny module of Siphoviridae	SACOL0329	5207..5404						
**phi-Q4ZCQ5**	putative bacteriophagal DUF2513 domain-containing protein	SACOL0330	5391..5771						
-	transcriptional regulator KMD47_gp11, SACOL0327 from Siphoviridae	SACOL0327/KMD47-gp11	5838..6083						
**phi-Q9B0H0**	hypothetical protein,	SACOL0328	6052..6417						
-	putative protein, replication module of Siphoviridae	MW1432	6472..6687						
-	putative protein	SARLGA251_07830						4892..5137	
-	hypothetical protein, SACOL0326	SACOL0326				5093..5317			4709..4933
-	hypothet. protein/phi PV83 orf 10-like protein from Siphoviridae	ST42eORF029				5359..5808			4973..5422
-	Uncharacterised protein	NCTC5663_00870							5370..5753
**phi-treG**	hypothet. protein/hypothet transcriptional regulator from Siphoviridae	NCTC5663_00871<br>ST42eORF072				 5825..6043		5208..5438 	5825..6055
-	phage DNA-binding protein/phage DNA-binding protein	LGA251-07790							3256..3510
-	putative protein	CA347_1953							3526..3729
-	hypothet. protein from Siphoviridae	CA347_1954							3726..3860
**phi-betR**	BetR domain protein/putative membrane protein from Siphoviridae	SAJRA307_20330		3524..3751	3524..3751				
-	conserved putative phage protein from Siphoviridae, MW1930, SAAV_2057, KMD47_gp09	MW1930, SAAV_2057, KMD47_gp09		3806..3949	3806..3949				
**phi-DUF0771**	DUF771 domain-containing protein from Siphoviridae	MW1928/SAAV_2056		4100..4420	4100..4420		3455..3775		
**phi-DUF1270**	phage protein, UG86_01455	UG86_01455	6988..7149	4417..4578	4417..4578				
**phi-dbp**	DNA-binding protein from Siphoviridae/putative protein, SACOL0333/SAPIG0346/phi-KMD47_gp14 phi-KMD47_gp14	SACOL0333	6712..6975						
**phi-DUF1270**	DUF1270 family protein, replication or lysogeny module of Siphoviridae. Caution, overlaps with hypothet-phage-protein_Sipho-0111_ROSA-ORF193, but in opposite orientation	SAS063<br>KMD47gp15<br>NCTC5663_00872				 6036..6197 	3772..3915 	 5422..5583 	 <br>6039..6206
-	hypothet. protein from Siphoviridae, overlaps with KMD47gp15 but opposite orientation	ROSA-ORF193	7008..7118	4437..4547	4437..4547	6056..6166		5442..5552	6059..6169
-	putative protein	SAOV_1961c							6207..6527
**phi-DUF1108**	lysogeny-assoc. DUF1108 family protein from Siphoviridae	MW1926/SAJRA307_03560/NCTC5663_00874				6299..6559		5674..5934	6619..6879
-	hypothet. protein from Siphoviridae, Gp157 family protein	SAOV_1958c				6573..7052		5949..6428	6894..7373
**phi-ssbP1**	single-stranded DNA-binding protein, replication module of Siphoviridae	SAJRA307_03590				7091..7690		6467..7066	7412..8011

Gene ID	Description	Locus tags	A5IT17-integr. prophage from V353 (CC816)	hlb-integr. prophage from JRA307, AP019751 (CC1)	hlb-integr. prophage from IMT39173 (CC1)	Phage 42e, NC_007052.1	hlb-integr. prophage from V641 (CC8115)	sufB-integr. prophage from IMT37083 (CC350)	sufB-integr. prophage from NCTC5663, LS483317 (CC350)
**phi-ssbP2**	single-stranded DNA-binding protein, replication module of Siphoviridae	C9J86-00295						7066..7494	
		SACOL0339				7690..8115			
		SAJRA307_03600							8011..8436
**phi-DUF0968**	putative HNHc nuclease/replication-assoc. DUF968 domain-containing protein/conserved putative phage protein, SACOL0340 from Siphoviridae	SARLGA251_07890				8129..8803			8450..9124
**-**	hypothet. protein from Siphoviridae	phi-92-ORF174							8540..8653
**phi-Q4ZAK4-rep**	putative bacteriophagal protein/replisome-organizer, replication module of Siphoviridae	SAST38_01154						8930..9691	
**phi-dnaC2**	Transposase-associated ATP/GTP binding protein, replication module of Siphoviridae	phi-55-ORF016						9704..10489	
**phi-dnaD2c**	DnaD&phage-associated-domain from Siphoviridae	NCTC5663_00879							9117..9881
**phi-dnaD2b**	DnaD domain protein from Siphoviridae	SAB1743c				8796..9551			
**phi-dbp**	phage protein/helicase loader	SACOL0342				9551..9907			9881..10237
**phi-dhlC-2a**	helicase 2/ 1242 nt, replication module of Siphoviridae	SACOL0343				9904..11145			10234..11475
**-**	putative phage related protein, SACOL0344	SACOL0344				11142..11357			11472..11687
**phi-trmB**	TrmB family regulatory protein from Siphoviridae	MW1429	7228..7551	4657..4980	4657..4980		4012..4335		
**-**	hypothet. protein from Siphoviridae MW1428, KMD47_gp17, SAR1544, SAPIG0348	MW1428	7566..7928	4995..5357	4995..5357		4350..4712		
**Q4ZCH8=DUF2800**	bacteriophagal protein from Siphoviridae	SAJRA307_20280/ MW1427	7925..9091	5354..6520	5354..6520		4709..5875		
**phi-treG**	hypothetical protein	SAJRA307_20270		6533..6793	6533..6793				
**phi-DUF2815**	conserved phage-associated protein/DUF2815 family protein/ssDNA annealing protein (KMD47_gp19)	SAJRA307_20260/ MW1926	9118..9675	6825..7382	6825..7382		5902..6459		
**phi-polA**	phage DNA polymerase from Siphoviridae, MW1425, KMD47_gi10, SAR1541	MW1425	9743..11695	7452..9404	7452..9404		6527..8479		
**phi-sri**	staphylococcal replication inhibitor, replication module of Siphoviridae	SAJRA307_03640						10486..10644	
**phi-rusA**	RusA family crossover junction endodeoxyribonuclease, replication module of Siphoviridae	SAOV_0297						10888..11295	
**phi-DUF1064**	DUF1064 domain-containing protein, replication module of Siphoviridae	SAOV_1085							11922..12326
		ST42eORF033				11593..11997			
**phi-DUF3113**	DUF3113 family protein, replication module of Siphoviridae	SACOL0348				12002..12187			12331..12516
		SAJRA307_03670						11295..11480	
		MW1424	11708..11893	9417..9602	9417..9602		8492..8677		
**phi-DUF3269**	DUF3269 family protein, SACOL0345, replication module of Siphoviridae	SACOL0345				11361..11582		10657..10878	11690..11911
**phi-xrep**	XRE family regulatory protein/helix-turn-helix transcriptional repressor, lysogeny module of Siphoviridae	SACOL0349				12188..12446			
		NCTC5663_00886							12517..12774
**phi-dbp**	putative DNA-binding protein/polymerase/phi PVL orf 50-like protein, replication module of Siphoviridae	SAAV_2043				12458..12817			12787..13146
		SaO11-00281/SAS0911	11917..12294	9626..10003	9626..10003		8701..9078		

Gene ID	Description	Locus tags	A5IT17-integr. prophage from V353 (CC816)	hlb-integr. prophage from JRA307, AP019751 (CC1)	hlb-integr. prophage from IMT39173 (CC1)	Phage 42e, NC_007052.1	hlb-integr. prophage from V641 (CC8115)	sufB-integr. prophage from IMT37083 (CC350)	sufB-integr. prophage from NCTC5663, LS483317 (CC350)
**phi-DUF3310**	DUF3310 domain-containing protein/hypothet nucleotide kinase from Siphoviridae	MS7_1486					9078..9335		
		MW1422							13146..13403
		SAS0912			10003..10257				
		SAJRA307_20220		10003..10260					
		SAPIG0353	12294..12548						
-	hypothet. protein from Siphoviridae	MW1421		10263..10463					13406..13606
**phi-DUF1270**	DUF1270 family protein, virulence-associated passenger protein, replication module of Siphoviridae	MW1420/ NCTC5663_00310	12554..12796	10478..10720	10263..10505				13621..13863
		SAOV_0301				12818..13066		12099..12347	
-	methylase	C9J86_14505					9535..10224		
-	putative protein, replication module of Siphoviridae	SACOL0353 /SAJRA307_03710		10734..11144	10519..10926		10239..10640	12361..12771	
-	hypothet. protein/GNAT family acetyltransferase from Siphoviridae	SAOV_0304			10926..11201		10640..10915	12771..13046	
-	hypothet. protein/GNAT family acetyltransferase from Siphoviridae	SAJRA307_03720		11144..11500					
**phi-DUF1024**	DUF1024 family protein/putative phage-related protein, replication module of Siphoviridae	SAJRA307_20170/ NCTC5663_00891	12811..13056	11493..11741	11194..11442	13081..13329	10908..11156	13039..13287	13878..14132
**phi-dut**	dUTP pyrophosphatase/Na/K ATPase replication module of Siphoviridae	phi-42e-ORF024			11417..11968	13304..13831			
		SACOL0357		11734..12267			11149..11676		
		SAS0919	13053..13589					13280..13816	14282..14818
-	hypothet. protein from Siphoviridae	ROSA-ORF092							14119..14289
-	putative protein	NCTC5663_00894						13853..14098	14855..15100
**phi-DUF1381**	DUF1381 domain-containing protein, transcriptional regulator, replication module of Siphoviridae	SACOL0358	13626..13832	12304..12510	12005..12211	13868..14074	11713..11919	14095..14301	15097..15303
-	hypothetical protein	KMD47-GP31						14289..14492	
-	hypothet. protein from Siphoviridae, SACOL0360	SACOL0360						14489..14692	
**phi-DUF1523**	replication-assoc. putative DUF1523 family protein from Siphoviridae	phi-42e-ORF038		12510..12863	12211..12564	14074..14427			
**phi-rinB**	transcriptional activator RinB, replication module of Siphoviridae	SACOL0361	13829..13981	12860..13012	12561..12713	14424..14576	11916..12068		
		SATW20-03570						14914..15087	15300..15473
-	hypothet. protein from Siphoviridae	CA347_1927						15088..15489	
-	putative protein	C9J86_14455						15419..15673	
-	hypothet. protein from Siphoviridae	ST42eORF198	14006..14107	13037..13138	12738..12839	14601..14702	12093..12194		
**Q4ZCN3=DUF1514**	hypothet. protein from Siphoviridae	MW1410	14049..14249	13080..13280	12781..12981	14644..14844	12136..12336		
**phi-virE**	phage virulence-associated protein E/DNA helicase	SAJRA307_20110	14301..16748	13332..15779	13033..15480	14896..15692	12388..14835		
-	hypothetical protein KMD47_gp36	KMD47_gp36		15842..15946	15543..15647	15755..15859			
-	hypothet. protein from Siphoviridae, rho-independent terminator of virE	phi-3A-ORF076/MW1407	16694..16894	15803..15925	15426..15626	15716..15838	14859..14981		

Gene ID	Description	Locus tags	A5IT17-integr. prophage from V353 (CC816)	hlb-integr. prophage from JRA307, AP019751 (CC1)	hlb-integr. prophage from IMT39173 (CC1)	Phage 42e, NC_007052.1	hlb-integr. prophage from V641 (CC8115)	sufB-integr. prophage from IMT37083 (CC350)	sufB-integr. prophage from NCTC5663, LS483317 (CC350)
-	hypothet. protein from Siphoviridae	phiST42eORF191	16851..16955	15882..15986	15583..15687	15795..15899	14938..15042		
**phi-nuc**	endonuclease /putative bacteriophagal protein from Siphoviridae	SAJRA307_20100/<br>KMD47_gp37	17089..17379	16120..16410	15821..16111	16033..16323	15176..15466		
**phi-dhlC-1**	DEAD/DEAH box helicase 1 1359nt from Siphoviridae	MW1405	17369..18727	16400..17758	16101..17459	16313..17671	15456..16814		
-	hypothet. protein from Siphoviridae	ST42eORF155	18516..18620	17547..17651	17248..17352	17460..17564	16603..16707		
**phi-rinM**	ORF between rinA and rinB, replication module of Siphoviridae	NCTC5663_00897							15477..15620
**phi-rinA**	RinA family transcriptional activator, replication module of Siphoviridae	SARLGA251_08070							15644..16066
**phi-Q4ZCF5**	hypothet. Protein/RinA-like transcriptional activator	SAJRA307_20080/<br>MW1404	18740..19177	17771..18208	17472..17909	17684..18121	16827..17264		
-	hypothetical protein	KMD47-gp40	19222..19347	18253..18378	17954..18079	18166..18291	17309..17434		
**phi-nuc-HNH**	HNH endonuclease family protein from Siphoviridae	SACOL0365/MW1403	19334..19648	18365..18679	18066..18380	18278..18592	17421..17735		
**phi-terS**	terminase small subunit, packaging module of Siphoviridae	MW1402	19775..20080	18807..19112	18508..18813	18711..19016	17863..18168		
		SARLGA251_08080						15877..16338	
		SAB1727c							16255..16749
**phi-terL**	terminase large subunit, packaging module of Siphoviridae	SAB1726c							16752..18041
		CA347_1925						16331..17554	
		MW1401	20070..21761	19102..20793	18803..20494	19006..20697	18158..19849		
**phi-port**	portal protein, packaging module of Siphoviridae	SACOL0368	21958..23004	20990..22036	20691..21737	20894..21940	20046..21092		
		NCTC5663_00901							18052..19587
		SARLGA251_08100						17557..18975	
**phi-micp/hmp**	minor capsid/head-morphogenesis-protein, head module of Siphoviridae	A7971-04170						18944..19894	
		SAB1724c/<br>NCTC5663_00902							19708..20589
		NCTC5663_00903							20662..20832
**phi-scaf=DUF4355**	DUF4355 domain-containing scaffold protein, head module of Siphoviridae	SARLGA251_08120						19990..20574	
		SAB1723c							20967..21557
**phi-clpP=Q5HIZ6**	Clp-protease/"head maturation protein" from Siphoviridae, packaging module of Siphoviridae	MW1399	22988..23761	22020..22793	21721..22494	21924..22697	21076..21848		
**phi-macp**	major capsid protein, head module of Siphoviridae	phi-69-ORF011							21571..22545
		SARLGA251_08130						20591..21505	
		SACOL0370	23773..24936	22805..23968	22506..23669	22709..23872	21860..23023		
-	hypothet. protein from Siphoviridae	ST42eORF172	24884..24994	23916..24026	23617..23727	23820..23930	22971..23081		
-	putative protein/phage terminase small subunit ?	SAB1721c							22567..22854
-	hypothet. protein from Siphoviridae	SARLGA251_08140						21517..21660	

Gene ID	Description	Locus tags	A5IT17-integr. prophage from V353 (CC816)	hlb-integr. prophage from JRA307, AP019751 (CC1)	hlb-integr. prophage from IMT39173 (CC1)	Phage 42e, NC_007052.1	hlb-integr. prophage from V641 (CC8115)	sufB-integr. prophage from IMT37083 (CC350)	sufB-integr. prophage from NCTC5663, LS483317 (CC350)
**phi-htcp**	phage head-tail connector protein	NCTC5663_00907							22863..23195
		M174_gp48						21667..22016	
		MW1397	25005..25283	24037..24315	23738..24016	23941..24219	23092..23370		
-	putative phage-related protein, tail protein ?	SAB1719c							23192..23494
-	Putative tail morphogenetic protein	SAB1718c/<br>NCTC5663_00909							23494..23841
-	putative protein, tail module of Siphoviridae	SAB1717c							23853..24236
-	hypothet. protein from Siphoviridae	phi-3A-ORF097	25123..25260	24155..24292	23856..23993	24059..24196	23210..23347		
-	hypothetical protein	MW1396	25295..25627	24327..24659	24028..24360	24231..24563	23382..23714		
-	putative phage protein	SAR1513/SACOL0373	25654..26025	24686..25057	24387..24758	24590..24961	23741..24112		
-	HK97 gp10 family phage protein	FIV54_08695						22350..22757	
**phi-DUF3168**	DUF3168 domain-containing hypothet. protein/"tail terminator" from Siphoviridae	SARLGA251_08180						22770..23195	
		MW1394	26026..26421	25058..25453	24759..25154	24962..25357	24113..24508		
**phi-matp**	putative tail protein	SARLGA251_08190						23196..23753	
		SAB1716c							24257..24838
**phi-matp1**	phi13 family major tail protein, tail module of Siphoviridae	MW1393	26456..27097	25488..26129	25189..25830	25392..26033	24543..25184		
-	hypothet. protein from Siphoviridae	ST42eORF083	26955..27155	25987..26187	25688..25888	25891..26091	25042..25242		
**phi-matp2**	major tail protein, tail module of Siphoviridae	SAPIG0382					25276..25731		
		MW1392	27189..27644	26221..26676	25922..26377	26125..26580			
**phi-DUF3647**	tail assembly chaperone, tail module of Siphoviridae	NCTC5663_00912							24900..25265
-	hypothet. protein, tail module of Siphoviridae	SAB1714c							25295..25639
-	hypothet. protein from Siphoviridae	phi-96-ORF039						23961..24326	
-	hypothetical protein,	SACOL0377	27702..28052	26734..27084	26435..26785	26638..26988	25789..26139		
-	hypothet. protein from Siphoviridae	CA347_1912						24506..24655	
-	hypothetical protein	SACOL0378/KMD47-gp53	28094..28252	27126..27284	26827..26985	27030..27188	26181..26339		
**phi-tmpM**	tail tape measure protein, tail module of Siphoviridae	MW1390/SACOL0379	28266..34466	27298..33474	26999..33175	27202..33402	26353..32553		
		SAHV_0896							25656..29123
		SARLGA251_08220						24659..27544	
**phi-Q4ZCS6**	holin or hypothet phage tail protein from Siphoviridae	MW1389	34466..35290	33474..34298	33175..33999	33402..34226	32553..33377		
		SARLGA251_08230						27559..28500	

Gene ID	Description	Locus tags	A5IT17-integr. prophage from V353 (CC816)	hlb-integr. prophage from JRA307, AP019751 (CC1)	hlb-integr. prophage from IMT39173 (CC1)	Phage 42e, NC_007052.1	hlb-integr. prophage from V641 (CC8115)	sufB-integr. prophage from IMT37083 (CC350)	sufB-integr. prophage from NCTC5663, LS483317 (CC350)
**phi-sitp**	tail protein/endopeptidase from Siphoviridae	SARLGA251_08240<br>MW1388<br>SAB1712c	 35299..36882	 34307..35890	 34008..35588	 34235..35818	 33386..34969	28511..30397	 <br>29136..30083
-	hypothetical protein,	SACOL0382	36882..37172	35890..36180	35588..35878	35818..36108	34969..35259		
**phi-mitp1**	minor structure protein, tail module of Siphoviridae/hypothetical phage protein	MW1386/SACOL0383<br>ROSA-ORF002	37188..39098	36196..38106	35894..37804	36124..38034	35275..37185	 30410..32308	
**phi-Q2FX67=pep**	phage minor structural protein/Endopeptidase, tail module of Siphoviridae	SAB1711c							30092..31993
-	Putative major teichoic acid biosynthesis protein C	NCTC5663_00917							32008..33918
-	hypothet. protein from Siphoviridae	phi-X2-ORF166						31401..31514	33011..33124
**phi-bppU**	minor tail protein/baseplate upper protein, tail module of Siphoviridae	SAB1709c<br>SACOL0384	 39098..40564	 38106..39572	 37804..39270	 38034..39500	 37185..38651	32308..34131	33918..35741
**phi-mitp2=DUF2977**	DUF2977 domain-containing protein/minor tail protein, tail module of Siphoviridae<br>*(truncated/incomplete)*	CA347_1906<br>SACOL0385/MW1384<br>SAPIG1496	 40564..40953	 39572..39961	 39270..39659	 39500..39889	 38651..39040	 <br>34380..34511	35741..36118
**phi-xldX**	XkdX family protein, tail module of Siphoviridae	SATW20-03820							36119..36301
**Q9MBN6=DUF2951**	DUF2951 domain-containing protein, tail module of Siphoviridae	C9J86_14345							36342..36641
**phi-hydr**	cell wall hydrolase, glucosaminidase/CHAP domain-containing protein, tail module of Siphoviridae	A7971-04265<br>NCTC5663_00922						35337..37031	 36778..38688
-	hypothet. protein from Siphoviridae	phi-85-ORF076							37223..37345
-	hypothet. protein from Siphoviridae	phi-X2-ORF207						36562..36663	38219..38320
**phi-bppU**	putative tailfiber-2/BppU baseplate upper protein, tail module of Siphoviridae	NCTC5663_00923						37044..37457	38701..39114
-	hypothet. protein from Siphoviridae	phi-53-ORF098						37118..37246	38775..38903
**phi-xldX**	XLdX family protein/putative phage protein TIGR01669	SACOL0386	40946..41110	39954..40118	39652..39816	39882..40046	39033..39197		
**Q9MBN6=DUF2951**	DUF2951 domain-containing protein, MW1382/SACOL0387, tail module of Siphoviridae	MW1382	41156..41455	40164..40463	39862..40161	40092..40391			
**phi-Q7A4M7**	putative bacteriophagal protein from Sipho-/Biseptimavirus	MW1890/SA1762					39631..40005		
**phi-txpA**	putative holin-like toxin/putative phage membrane protein	MW1888	41653..41784	40661..40792	40359..40490	40589..40720	40194..40325		
-	putative protein	SAA6159-03495	41837..41944						
**phi-holA**	holin from Sipho-/Biseptimavirus	SAB0780	42017..42250	41015..41248	40713..40946	40943..41176	40548..40781	37535..37768	39192..39425
**phi-amidase**	CHAP domain-containing protein/endolysin/amidase, lysis module of Siphoviridae	MW1886	42262..43017	41260..42014	40958..41713	41188..41943	40793..41548	37780..38535	39437..40192
**sak_phi-42e**	putative horse-associated staphylokinase	SAJRA307_19810/<br>ST42eORF010	43205..44299	42202..43296	41901..42995	42131..43225	41736..42830	38723..39817	40380..41474
-	putative protein	SAB1702c		43393..43648					41571..41827

Gene ID	Description	Locus tags	A5IT17-integr. prophage from V353 (CC816)	hlb-integr. prophage from JRA307, AP019751 (CC1)	hlb-integr. prophage from IMT39173 (CC1)	Phage 42e, NC_007052.1	hlb-integr. prophage from V641 (CC8115)	sufB-integr. prophage from IMT37083 (CC350)	sufB-integr. prophage from NCTC5663, LS483317 (CC350)
**phi-pdp_SAU**	phage defence protein of *S. aureus* as described in mbio.02490-22	SAJRA307_19800		43748..44584					
**-**	hypothet. protein from Siphoviridae	ST42eORF161	44502..44603		43201..43302	43431..43532	43037..43138		
**phi-DUF3467**	hypothet. protein from Siphoviridae, DUF3467 domain-containing protein	ST42eORF048			43423..43734	43653..43964	43259..43570		
**-**	hypothet. protein from Siphoviridae	ST42eORF037			43721..44104	43951..44334	43557..43940		
**-**	putative protein	NCTC5663_00927							41930..42349
**-**	putative protein	NCTC5663_00928							42363..43535
**phi-guaC**	dehydrogenase/GMP reductase	ST42eORF012		44940..45917	44317..45294	44547..45524	44153..45130		
**Q2YVE2**	putative protein	SAB0269	44716..45303						
**Q2YVE1**	putative protein	SAB0270	45303..45755						
**-**	putative protein	SAOV_1907						40338..40517	
**-**	putative protein	SAPIG0398/1490						40539..41066	
**-**	putative protein	SAPIG0399						41056..41454	
**-**	putative protein	EBI08-04040						41716..41945	43806..44035
